# Discovering highly selective and diverse PPAR-delta agonists by ligand based machine learning and structural modeling

**DOI:** 10.1038/s41598-019-38508-8

**Published:** 2019-01-31

**Authors:** Benny Da’adoosh, David Marcus, Anwar Rayan, Fred King, Jianwei Che, Amiram Goldblum

**Affiliations:** 1 Molecular Modeling Laboratory, Institute for Drug Research, The Hebrew University of Jerusalem, Jerusalem, 91120 Israel; 2 Institute of Applied Research, Galilee Society, Shefa-Amr, 20200 Israel; 3 Drug Discovery Informatics Lab, Qasemi-Research Center, Al-Qasemi Academic College, Baka El-Garbiah, 30100 Israel; 4 Genomics Institute of the Novartis Research Foundation, 10675 John Jay Hopkins Dr., San Diego, CA 92121 USA; 5 Department of Chem. & Biochem., University of California at San Diego, La Jolla, CA 92037 USA

## Abstract

PPAR-δ agonists are known to enhance fatty acid metabolism, preserving glucose and physical endurance and are suggested as candidates for treating metabolic diseases. None have reached the clinic yet. Our Machine Learning algorithm called “Iterative Stochastic Elimination” (ISE) was applied to construct a ligand-based multi-filter ranking model to distinguish between confirmed PPAR-δ agonists and random molecules. Virtual screening of 1.56 million molecules by this model picked ~2500 top ranking molecules. Subsequent docking to PPAR-δ structures was mainly evaluated by geometric analysis of the docking poses rather than by energy criteria, leading to a set of 306 molecules that were sent for testing *in vitro*. Out of those, 13 molecules were found as potential PPAR-δ agonist leads with EC_50_ between 4–19 nM and 14 others with EC_50_ below 10 µM. Most of the nanomolar agonists were found to be highly selective for PPAR-δ and are structurally different than agonists used for model building.

## Introduction

During the course of drug development, good binders (i.e., inhibitors, agonists) to biological targets are sometimes not useful as drug candidates due to severe adverse effects^1,2^. It is therefore customary to further explore the chemical space around these binders hoping to overcome their undesirable outcome by searching for close analogues of their scaffolds^3^. However, this approach does not guarantee success.

In this paper we present results of our in-house Machine Learning algorithm, Iterative Stochastic Elimination (ISE)^4^, which is an expert algorithm for predicting novel bioactives with highly diverse structures, combined with docking and geometry filters.

Peroxisome Proliferator-Activated Receptor-δ (PPAR-δ) has a well-known effective agonist, GW501516, that was abandoned as a drug due to promoting cancer in preclinical animal testing^5,6^. GW0742, has a similar scaffold and has also been associated with adverse effects^6^. However the search for a drug that acts at PPAR-δ continues^7,8^. We discovered 27 novel agonists of PPAR-δ (13 with low nanomolar EC_50_) which have diverse scaffolds compared to previously known agonists and vis-à-vis each other.

Peroxisome Proliferator-Activated Receptors (PPARs) are a subgroup of the nuclear hormone receptor family. Its members, PPAR-α, PPAR-γ and PPAR-δ (known also as PPAR-β), are ligand-activated transcription factors^9–12^. PPAR-α is expressed in muscle and heart tissues, but mainly in the liver. PPAR-γ acts as a master regulator of adipocyte formation. PPAR-δ is expressed in many tissues, but at low levels in the liver^13^.

Like other transcription factors, PPARs form heteromers with retinoid X receptor (RXR) and additional co-activator proteins^14^. By binding PPARs to endogenous ligands such as fatty acids, eicosanoids and oxysterols, these ligand-activated transcription factors function as fat sensors^13^, and maintain lipid and glucose homeostasis that are important in preventing cancer, diabetes, obesity and atherosclerosis^14^.

PPAR structures have two domains: The N-terminal is a DNA-binding domain, with a dual zinc-finger motif; The C-terminal is a ligand binding domain (LBD), which consists of 12 α-helices and 3 β-strands^15^. These secondary elements form a large hydrophobic binding cavity of 1300 Å^3^. The C-terminal α-helix is AF-2 (Activation function helix-2), which is involved in recruitment of co-activators^16^. Binding of a co-activator to a key tyrosine residue on this helix stabilizes the active conformation. Since the LBDs of PPARs are highly similar, some of the agonists are dual or “pan agonists” and bind to all three PPARs^14^.

Both pan and isoform specific PPAR agonists can be beneficial under different scenarios. PPAR agonist drugs enhance PPAR activities. Fenofibrate (Tricor^TM^) and Bezafibrate (Bezalip^TM^) are mostly known as PPAR-α agonists for treating dyslipidemia by reducing triglycerides (TG) and free fatty acids (FFA) and increasing the levels of high-density lipoproteins (HDL). PPAR-γ’s agonists are Glitazones, such as Rosiglitazone (Avandia^TM^) and Pioglitazone (Actos^TM^)^17^. They are used in the treatment of type 2 diabetes by improving insulin sensitivity, reducing plasma glucose, TG and FFA as well as increasing HDL.

PPAR-δ may serve as a promising target as its agonist (GW501516) has known beneficial effects on obesity, on insulin resistance, and on reduction of plasma glucose in rodent models of type 2 diabetes. In addition, studies on obese primates suggest that this agonist decreases low-density lipoprotein (LDL), TG and insulin, and increases HDL. In sedentary human volunteers, this agonist prevented the decrease of HDL-c and apoA-1 levels by reducing of serum TGs^17^. It has also been suggested recently that PPAR-δ agonists promote exercise endurance by preserving glucose^18^. It also has the potential to treat atherogenic dyslipidemia and non-alcoholic fatty liver disease^19^. Other effects such as its role in epidermis repair by keratinocyte proliferation^20,21^, contribution to the resolution of inflammation after gut ischemia/reperfusion injury^22^ and reducing lung inflammation^23^, have also been described.

There are, however, no PPAR-δ agonists in clinical use yet. Recently a new structure-based computational tool was developed to search for PPAR-δ agonists, but no novel agonist has been reported^24^. We have therefore begun a search for novel and yet unknown chemical entities which may form the basis for such targeting, by employing computational methods including our in house computational classification algorithm, ISE^4^.

ISE has been presented in several publications in recent years^25–27^. It has been used mostly to produce classification models based on previously published effects of ligands. A combination of ISE, docking and geometry filters was used here to create a highly efficient model, which successfully distinguishes between known agonists of PPAR-δ and random molecules (presumed to be inactive).

**Figure 1:**
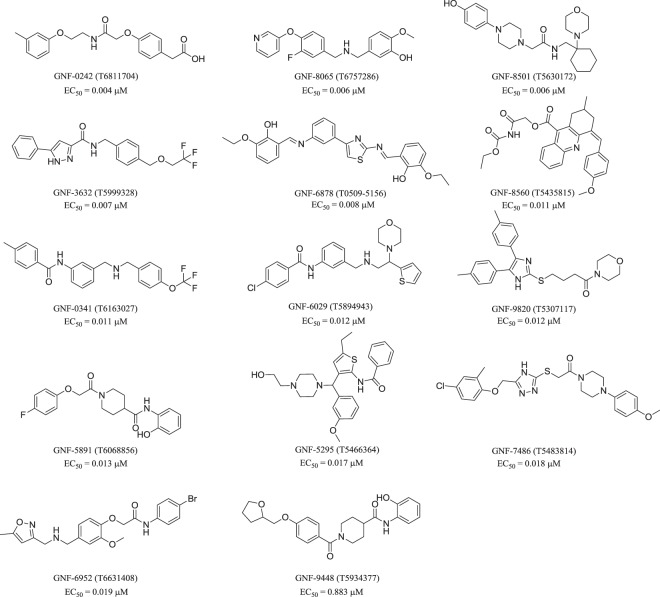
Structures and experimental results of lead agonists of PPAR-δ discovered in this study. For ZINC id and SMI codes – see Table S1.

The ENAMINE library^28^ composed of 1.56 million commercially available molecules has been screened and ranked by the ISE model, and top ranking molecules were docked to PPAR-δ. Molecules that showed potential activity by ISE ranking and by docking calculations were purchased for *in vitro* testing. Out of 306 molecules submitted to testing, nearly 9% were found to have good to excellent binding affinities: 13 molecules have EC_50_ in the low nanomolar range (4–19 nM) and 14 others have EC_50_ < 10 µM (one of those with EC_50_ = 883 nm). The top active molecules with EC_50_ < 1 µM are presented in Fig. 1.

## Results

### PPAR-δ agonists dataset

Agonists (789) were collected from different literature sources including CHEMBL^29^ and WOMBAT^30^. The range of their agonist activities (EC_50_) was 0.03–1000 nM. Figures S1–S3 present some characteristics of those 789 PPAR-δ agonists. They partially obey Lipinski’s Rule of Five (ROF) for Oral availability of drugs^31^ and Oprea’s rules for “Lead like molecules”^30^ (63% and 25% of the ligands, respectively). The violations are mainly due to high lipophilicity and high molecular weight of PPAR-δ agonists. The range of molecular weights is between 300–600 Daltons. There are no hydrophilic ligands, and most are hydrophobic with clogP value above 3. However, their functional groups have the potential to form strong specific interactions – hydrogen bonds and electrostatic interactions.

This dataset was divided in two, with one-half (394 molecules) serving as training set, and the others (395) as test set. Typically, active molecules are prone to be highly similar due to their shared synthetic procedures when applied for hit and lead optimization, often using the same scaffolds and chemical series. To avoid such bias, the diversity of the training set of actives needs to be increased, by limiting the similarity within the set. On the other hand, a demand for highly diverse molecules might exclude too many potential ones. Figure S4 presents the numbers of active molecules that remain at each “cutting edge” of Tanimoto imilarity scores (T)^32^ in both the training and the test set. The T = 0.8 threshold was found to present a fair balance between having diverse molecules and having enough molecules. Thus, 129 active molecules were excluded from the training set for model construction and 265 actives remained.

### Five thousand molecules were collected randomly from the ENAMINE database to represent the set of “inactives”

The assumption is that, statistically, most of those randomly chosen molecules are inactive although they have not been confirmed as such. The set size of inactives is a compromise: it should represent accurately the properties’ space of the inactives but the number should not be too large, which might extend computation time and risk considerable bias (see discussion). Nearly 98% of these molecules have Tanimoto values of <0.4 to others. Those inactives were picked by limiting some of their properties based on the idea of an “applicability domain”^33^, as described in the methods.

### A model for identifying of PPAR-δ agonists was created by Iterative Stochastic Elimination (ISE)

A flowchart of the ISE process is presented in Fig. S5. The ISE modeling was performed with a training set of 5265 molecules, for classifying the 265 actives against the class of 5000 inactives. We generated sixty-eight unique filters, with 4 descriptors each, that distinguish best between PPAR-δ agonists and the inactive molecules. The top filter had an MCC value (Matthews Correlation Coefficient)^34^ of 0.97 (98% TP, 99% TN) and the least classifying filter had an MCC of 0.755 (91% TP, 84% TN). Filters have been clustered to ensure dissimilarity >1% between filters. That is, if two filters are identical in the numbers of actives/inactives to the extent of >99%, the one with lower MCC is discarded. For details about the compositions of the filters – and for the meaning of descriptors see Table S2.

### The ISE model was validated by the test set

The test set contained 395 known agonists and 10,000 “newly picked” inactives from ENAMINE. It was used in order to test the sixty-eight unique filters (the final model). The model produced a molecular bioactivity index (MBI) for each molecule in the test set. The MBI score^4^ is a result of the number of filters successfully passed (by having properties that fully fit the 4 descriptor ranges of a filter), which add their TP/FP value to the successfully passed molecule, while missing any filter (if one non-fitting descriptor’s value of any filter is found in a molecule) reduces the MBI score by TP/FP of that filter.

**Figure 2:**
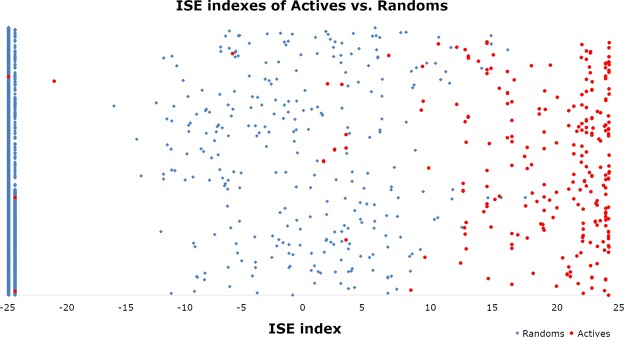
MBI indexes according to our PPAR-δ model for the full test set, consisting of actives + inactives, (10395 molecules in total).

The model was highly efficient for scoring bioactivity on PPAR-δ. More than 96% of the actives in the test set were captured at the top 1% of the scores (10,395 molecules, including 10,000 random molecules). The area under the ROC is above 0.98, revealing a highly accurate and efficient model. See the enrichment plots in Fig. S6 and ROC curve plots in Fig. S7. In Fig. 2, the X-axis indicates MBI values (between −25 and +25) for the molecules that are simply counted on the Y-Axis, with no particular order. The relatively small number of filters is responsible for the fact that there are many very negative scores (mostly of TN) while there are only few molecules with MBI scores between −25 and −15. Larger MBI values are associated mostly with the true positives. In Table 1, each column represents an index (MBI) border between molecules considered to be positives (which are with higher MBI values) and those considered to be negatives, which have lower MBI values. As we know which ones, on each side, are actives or are assumed inactives, it is easy to compute the 6 values of each column. It is clear that enrichments are much larger at higher MBI values, while the numbers of TP become smaller, and so does the number of TN. However, MCC values do not change linearly, and are maximal around the MBI value of 3. As much as there is no dramatic difference from +6 to +10, we deal in the discussion with our decision to pick from screening only molecules with MBI of 10 and greater.

**Table 1:** MCC scores and Enrichment Factor for the MBI.

MBI Border						
	−3.0	+3.0	+6.0	+9.0	+10.0	+13.0
TN	6048	9682	9876	9969	9983	10000
FN	12	81	110	150	211	277
TP	383	314	285	245	184	118
FP	3952	318	124	31	17	0
Enrichment*	2.5	25	58	199	268	—
MCC	0.616	0.774	0.736	0.665	0.547	0.419

A threshold of MBI ≥ 10.0 was used to construct the first focused library.

^*^Calculated based on the assumption that none of the ENAMINE DB chemicals is active on PPAR delta.

### The ENAMINE database of 1.56 million molecules was screened through the model

Only 2,491 molecules achieved an MBI score >10. These molecules were used in the next step of docking and then for the selection for *in vitro* tests. Table S3 presents the number of the commercial molecules in ENAMINE, which have indexes over various thresholds. None of the 2,491 molecules were examined previously for PPAR-δ binding. The top 2,491 commercial molecules that got indexes above +10 in the ISE model were subject to docking with OpenEye’s FRED^35^. Finally, 306 top molecules were purchased for *in vitro* binding experiments as described below.

### Five PDB complexes were collected and used to define the most important residues for docking

Those PPAR-δ complexes (1GWX^36^, 3GWX^36^, 3D5F, 3ET2^37^ & 3GZ9^13^) were collected from the PDB (see methods).

We used the 5 complexes in order to construct a list of interactions of protein residues with the crystallized ligands, shown in Fig. S8. Nearly 30 residues in those complexes have one or more close connections to their ligands by distance criteria that are described in the methods. The maximum distance for VdW interactions is 3.9 Å. Cys285, Thr288, Thr289, His323, Leu330, Ile364, His449, Met453, Leu469 and Tyr473 interact with more than 3 different ligands or create H-bonds with one of them, and these residues were defined as “important”. In addition, His323, His449 and Tyr473 consistently create a Hydrogen bond with all the ligands and so we define them as “crucial to H-bonds”.

### Two PDB complexes were found optimal for docking

The Ramachandran plots^38^ of 3GZ9 and 3D5F do not have any outliers, while those of 3ET2, 3GWX and 1GWX have 2, 5 and 10 outliers, respectively (Supporting Information Fig. S9). In re-docking, we test how well the original ligand of a complex is predicted by the docking protocol. Except for the case of 3ET2, all the other ligands fulfilled the criteria described in the methods. Geometrical data, as well as energy score, for each selected pose in the re-docking is presented in Table S4.

Since we search for novel agonists, a crucial test of the ability of the docking algorithm and of a specific crystal complex is that of distinguishing between two sets of molecules, the known agonists and the inactives. The measure for success of discrimination between the sets is MCC. One hundred thirty-five molecules were picked out of the 789 agonists (each of them has Tanimoto value <0.7 to the others) and were tested on each one of the complexes. The criteria for success in docking were the same as in the re-docking validation test. Two chains (3D5Fa and 3D5Fb) of the same crystal complex and one complex, 3GZ9 identified the largest numbers of true positives (more than 100 out of the 135. See Table S5). The data regarding resolutions and Ramachandran Plots support these results, as these complexes have better resolution and Ramachandran Plot. Out of 1000 Random molecules (different from the set that was described above – see applicability domain in Table S6), only 179, 130 and 143 were docked, respectively. Therefore, MCC of 0.68–0.69 were calculated for each complex.

### The 2491 molecules with top MBI scores were screened by docking to the three selected chains

The best results were picked on the basis of “voting”: 335 ISE hits were successfully docked to all three PPAR-δ complexes, 349 were successful in only two chains and 489 were successful in one only. The 335 hits that were successful in docking to all the 3 selected PPAR-δ complexes are highly diverse in comparison to the 394 agonists of the training set. Tanimoto index <0.3 is found for 318 molecules to all the others, while the rest 17 molecules have Tanimoto index <0.4. Among these 318 hits, 306 were available for purchasing, and sent to the Genomics Institute of Novartis Research Foundation (GNF) for *in vitro* experiments.

### EC_50_ values (for activation) of the 306 candidates were determined for each of the three human PPARs

GW501516 was used as the positive control for hPPAR-δ with EC_50_ value of 0.001 µM for hPPAR-δ (EC_50_ values of 0.704 and 0.839 µM for hPPAR-α and hPPAR-γ, respectively)^39^. GW7647 was used as the positive control for hPPAR-α with EC_50_ value of 0.003 µM for hPPAR-α (EC_50_ values of 0.974 and 0.85 µM for hPPAR-δ and hPPAR-γ, respectively)^39^. GW1929 was used as the positive control for hPPAR-γ with EC_50_ value of 0.013 µM for hPPAR-γ (EC_50_ values of 1 µM for hPPAR-δ and hPPAR-α)^40^.

**Figure 3:**
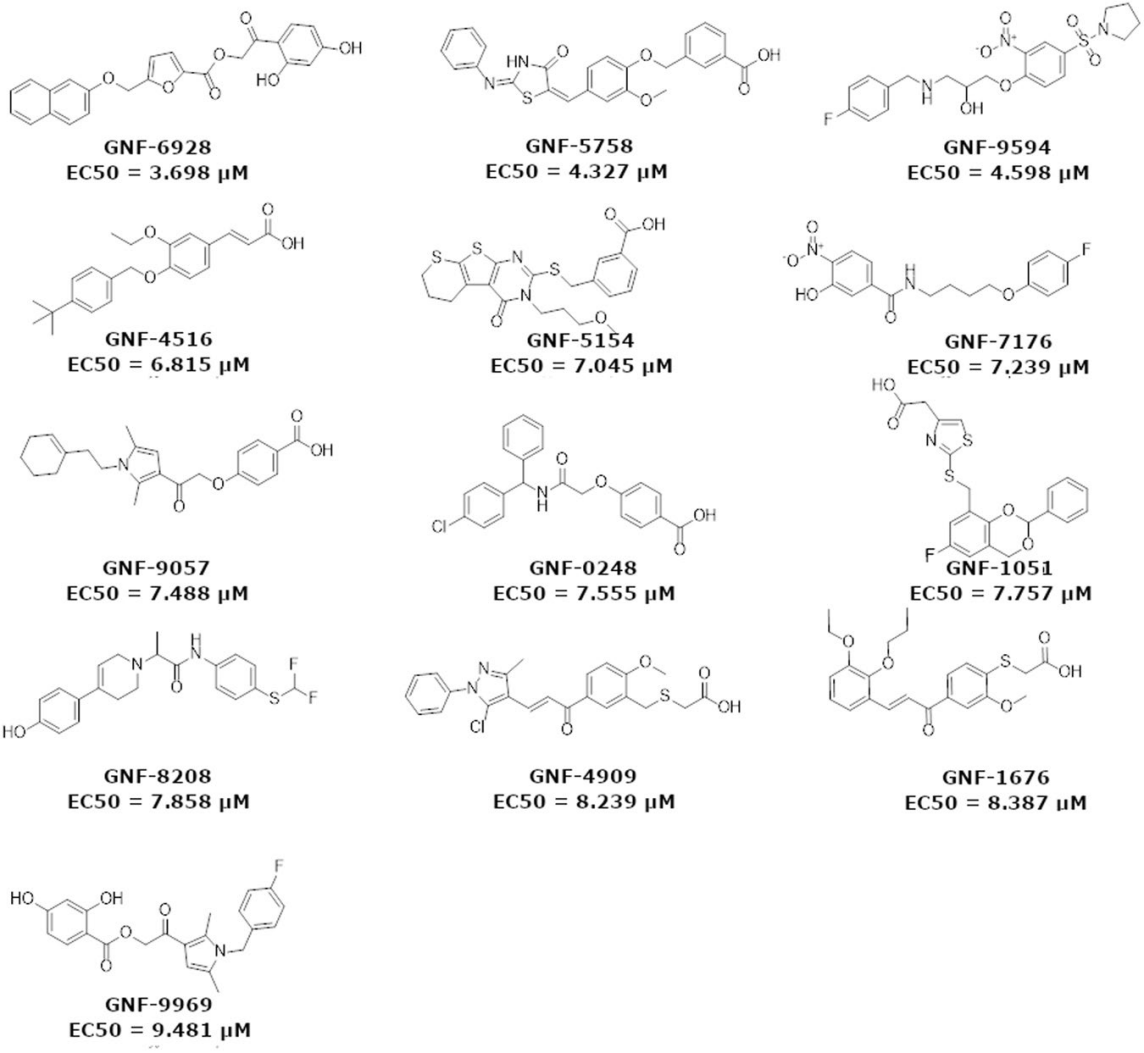
Structures and EC_50_ values of novel agonist hits (EC_50_ > 1 µM) of PPAR-δ, discovered in this study. For ZINC id and SMI codes – see Table S1.

The *in vitro* results were categorized into 4 main classes: 1) agonists of hPPAR-δ with highest affinity (EC_50_ < 1 µM), 2) agonists of hPPAR-δ with low affinity (EC_50_ > 1 µM), 3) non-agonists of hPPAR-δ but agonists of other hPPARs and 4) non-agonists of hPPARs. The first class has 14 hits (Table 2 and Fig. 1). Only one of them is a “pan-agonist” hitting all three targets (GNF-8560), two of them are agonists of hPPAR-δ and hPPAR-α only (GNF-3632 & GNF-6952), and two of them are agonists of hPPAR-δ and hPPAR-γ only (GNF-5295 & GNF-0341). All the remaining 8 agonists are highly selective for hPPAR-δ. The second class has 13 hits (Table 3 and Fig. 3). Four of them are selective to hPPAR-δ. The third class has 64 hits (Table S7 and Fig. S10). Most of them have low affinities. Only 3 of them have EC_50_ < 1 µM for hPPAR-γ, and the best (GNF-6635) has EC_50_ = 0.277 µM and is not active on the other two targets. The other two (GNF-7017 & GNF-1165) have EC_50_ < 1 µM for hPPAR-γ, and EC_50_ > 1 µM for hPPAR-α.

**Table 2:** EC_50_ values of three reference agonists and of top 14 molecules with strongest affinities (EC_50_ < 1 μM) for hPPAR-δ. EC_50_ values for the other hPPARs are presented.

EC_50_ (μM)			
Name	hPPAR-δ	hPPAR-α	hPPAR-γ
GW501516	0.001	0.704	0.839
GW7647	0.974	0.003	0.85
GW1929	1	1	0.013
GNF-0242	0.004	>10	>10
GNF-8065	0.006	>10	>10
GNF-8501	0.006	>10	>10
GNF-3632	0.007	5.477	>10
GNF-6878	0.008	>10	>10
GNF-8560	0.011	1.406	3.525
GNF-0341	0.011	>10	1.258
GNF-6029	0.012	>10	>10
GNF-9820	0.012	>10	>10
GNF-5891	0.013	>10	7.279
GNF-5295	0.017	>10	>10
GNF-7486	0.018	>10	>10
GNF-6952	0.019	7.559	>10
GNF-9448	0.883	7.061	0.937

Molecular structures are shown in Fig. 1.

**Table 3:** EC_50_ values of 13 molecules with lower affinities (EC_50_ > 1 µM) for hPPAR-δ.

EC_50_ (μM)			
Name	hPPAR-δ	hPPAR-α	hPPAR-γ
GW501516	0.001	0.704	0.839
GW7647	0.974	0.003	0.85
GW1929	1	1	0.013
GNF-6928	3.698	6.904	3.784
GNF-5758	4.327	6.930	9.732
GNF-9594	4.598	>10	>10
GNF-4516	6.815	2.327	>10
GNF-5154	7.045	>10	>10
GNF-7176	7.239	>10	>10
GNF-9057	7.488	4.633	8.595
GNF-0248	7.555	7.036	>10
GNF-1051	7.757	7.331	>10
GNF-8208	7.858	>10	>10
GNF-4909	8.239	0.825	1.834
GNF-1676	8.387	6.195	1.287
GNF-9969	9.481	6.937	1.010

EC_50_ values for the other hPPARs are presented. For molecular structures – see Fig. 3.

### All the new active scaffolds are diverse both with respect to the “learning set” as well as with respect to each other

One of the advantages of our methods is due to the representation of molecules as sets of physico-chemical properties and not as structural fragments. This has been repeatedly shown to lead to the discovery of novel scaffolds in our previous projects^41,42^. Tanimoto index was used in the training set to eliminate active molecules that are similar at a level of Tanimoto index ~0.8 and greater.

We compared the novel agonists to the training and the test set of actives. It is a matrix of Tanimoto values (each row is a known agonist while each column is a newly discovered agonist). The highest value in this matrix is 0.57, indicating that novel agonists are very different from the known 789 agonists from the ChEMBL and WOMBAT databases. Another interesting result is the diversity among the 27 newly discovered agonists. None of the values is greater that T = 0.7. Out of 351 Tanimoto values, only 8 are with T > 0.4, again indicating large diversity of the novel agonists.

**Figure 4:**
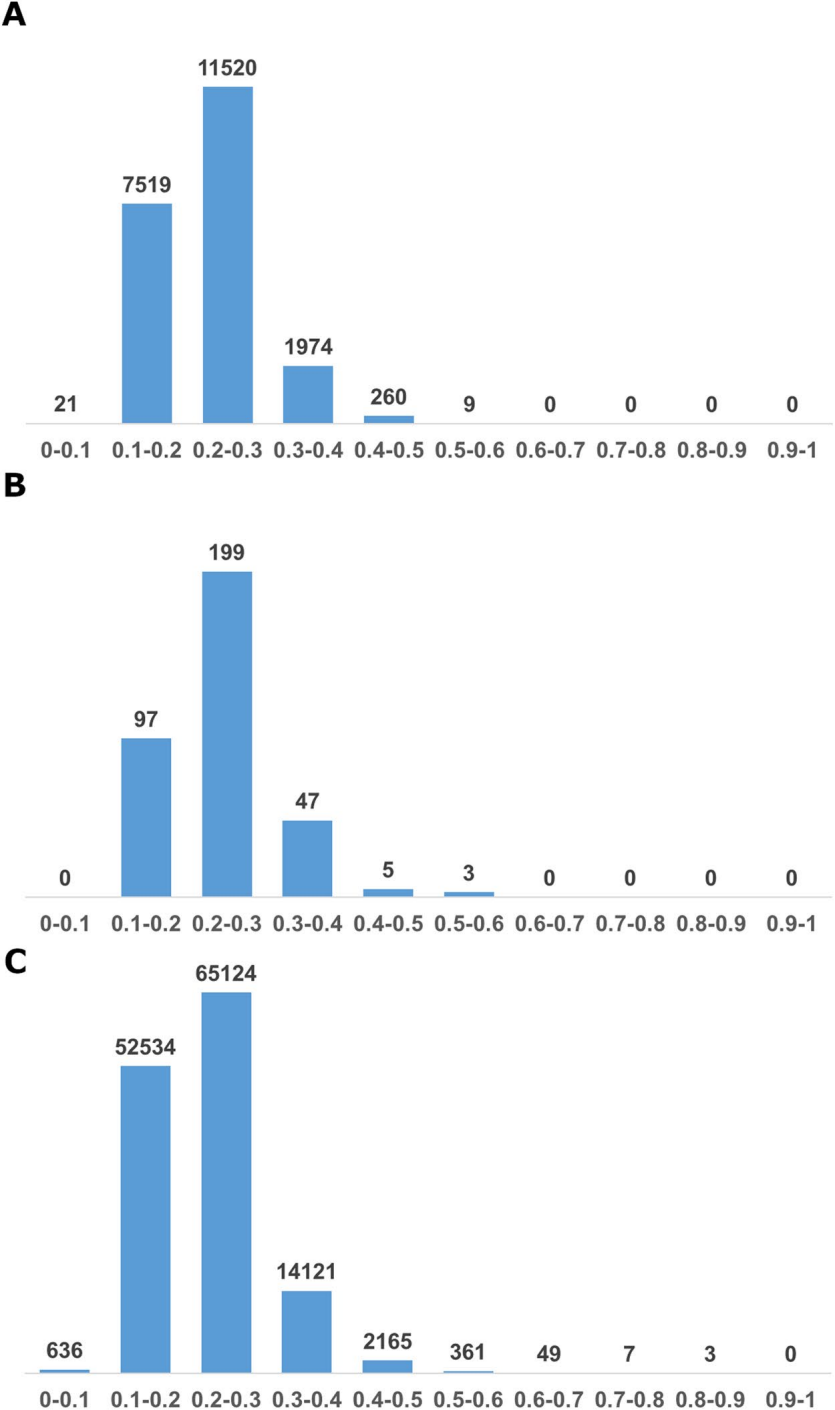
Distribution of Tanimoto values for the novel agonists. (**A**) Novel agonists were compared to all known agonists in training and test sets. (**B**) Comparison of novel agonists among themselves. (**C**) Novel agonists were compared to the random set.

Figure 4 presents the distribution of Tanimoto values for the three comparisons – the 789 known agonist set vs. novel agonists (Fig. 4A) and novel agonists among themselves (Fig. 4B). The third comparison is between the discovered 27 agonists and the randomly picked set of 5000. Only 10 out of the 135000 have T values above 0.7 (See Fig. 4C).

Recently, Wu et. al. discovered 16 new agonists^43^, all of which have high MBI scores of 13–19 in our models (Table S8), so the ISE model could identify them as agonists had they been in the dataset of the virtual screening. These agonists have no similarity to our novel agonists (see Fig. S11). We searched for similar molecules of these agonists in the ENAMINE catalog (in order to check if our model missed potential agonists). Only four molecules out of the 1.56 million have similarities of Tanimoto value > 0.7 to compound 1 (T0517-7230 & T5405641 have 0.74; T5681815 & T5999586 have 0.71). None of the ENAMINE molecules is similar to the other agonists.

Scaffolds of these new agonists are based on the substrate, and are similar in most of the cases. Figure S11b shows that all have Tanimoto values >0.6 to each other (and in most cases it is >0.7). Furthermore, 4 of these agonists (Compounds 1, 12, 13 & 14) have Tanimoto values > 0.7 to the 789 known agonists (our training and test sets), while in the case of our novel agonists none has similarity to Tanimoto value > 0.7 to the known agonists.

Finally, we used the Tanimoto index to compare our novel agonists vs. all molecules that are known in BindingDB^44^ or predicted agonists in ZINC15^45^. None of our agonists were found to have high similarity (>0.7) to any of the known or predicted ones.

## Discussion and Conclusions

**Figure 5:**
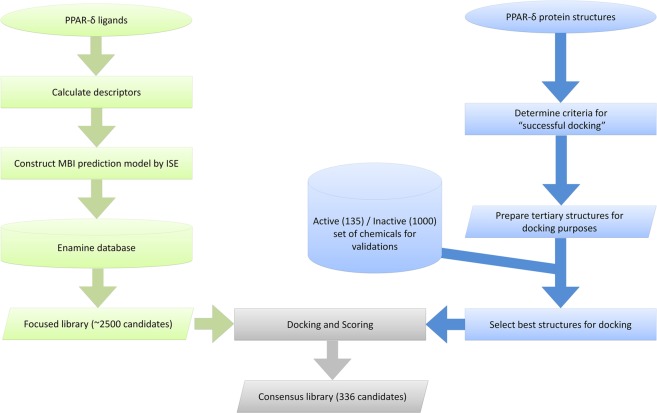
Flowchart of our ligand- and structure-based combined approach to prepare a focused library of PPAR-δ bioactive candidates.

By using a combination of two computational methods (ISE + docking) we discovered 27 novel hit and lead agonists of PPAR-δ out of which 13 are highly selective low nanomolar activators with EC_50_ < 19 nM. The selectivity was obtained, however, without any direct study and prediction for the other PPAR subtypes. Other 14 molecules are “hits” with EC_50_ < 10 μM. Figure 5 is a flowchart that illustrates how we combined Ligand-based and Structure-based strategies.

Our ISE algorithm constructed a model of 68 filters (each is an ensemble of 4 ranges of physico-chemical properties) which were used for screening those highly active molecules. By passing or failing to pass filters, molecules accumulate the scores from single filters to the final MBI score. Therefore, despite the fact that each filter can only classify molecules in a binary fashion, the set of filters allows to shift from a qualitative classification to a relatively quantitative one. Contrary to QSAR equations with several variables, it would be unfeasible for an organic chemist to consider the contents of 68 filters in parallel to suggest synthesis of new candidates.

The results of the ISE modeling in combination with geometry filters of docking results enabled the discovery of new ligands with novel scaffolds for a known target, as shown by Tanimoto values of the 27 new PPAR-δ agonists, compared to the previously known agonists, which were used to construct the model. The discovered agonists (hits and leads) also form a diverse set. This is a result of the modeling process being based on initially non-structural 2D representation of molecules followed by 3D structural criteria by docking.

While other methods are based on the scaffolds of known actives^43^, which frequently results in “me too” or “follow-on” drugs^3^, the use of physico-chemical properties to characterize molecules abandons the structural portrayal and gains the ability to avoid similarity to known agonists, in our case. This is a clear advantage of ISE modeling in comparison to those other methods.

Are the discoveries of hits and leads of PPAR-δ a pure result of modeling or is there a random component?

What is the probability for discovering active molecules by chance from a database of 1.56 million molecules that was virtually screened by the ISE model?

The rate of discovery in “wet” high throughput screening is typically 1:1000^46^ for finding hits, defined as having activity in the micromolar range of 1–10 µM while leads, with an activity in the nanomolar range, are much less abundant. Assuming that a similar rate is expected in virtual screening, more than 1500 hits should be found among 1.56 million molecules. Picking 306 molecules out of 1.56 million, and discovering 27 hits and leads, suggests a substantial enrichment factor (EF). Even if we assume that only hits were discovered (while among those 27, there are 13 leads with 4–19 nM EC_50_) the EF value is ~88 = (27/306) / (1560/1560000). As nearly half are lead molecules, it is expected that EF would be much greater.

We have chosen the number of molecules to be purchased as a compromise between two considerations: 1) large values of TP/FP and 2) picking enough molecules to guarantee higher chances to find active molecules. Combining those two is the reason for using the model index of +10 and above, with 184 (out of 265) True Positives and 17 (out of 5000) False Positives.

Could we identify a larger number of actives by modeling and screening? What is the total number of actives expected to be found in a large database of 1.56 million molecules? If we assume that all the known agonists (about 800 in ChEMBL) that were already reported in the literature are among the ~30 million commercially available molecules and are equally spread between the companies, there may be 40–50 actives in the database of ~1.5 million that we used for screening. This also sheds light on another issue. It is frequently suspected that the “inactives” contain also “actives”, and it is questioned how many of the randomly picked “inactives” could be actives, i.e., “false inactives”. In the present case, if about 50 molecules could be discovered out of 1.56 million (we managed to find about half of that), picking randomly 10000 molecules (as we did in the test set) may end in a single active molecule at most, one that is wrongly considered to be inactive.

The “inactives” used in the modeling process are randomly picked from a database of chemicals, based on the assumption that they are not active. Had we had reports on PPAR-δ testing that found molecules with no agonist activity, it would be better to use such a resource, but literature reports of inactive molecules in any experiment are quite rare. So even those randomly picked molecules, which get high indexes and are characterized as FPs could be, at least some of them, hits or even leads. The assumption of inactivity for those randomly picked molecules is still valid, because only 27 molecules were discovered out of 1.56 million, constituting way less than 0.1% actives.

Molecules, which interact with a specific target, may frequently be part of a set that has some similarity of molecular properties. Rather than using structural properties of the ligand, which may imply interactions and energies, we focus on properties that can help to bring a ligand to the target, i.e., pharmacokinetic relevant ones, such as the Lipinski Rule of 5 (RO5) properties^31^. If we find such a collection of properties, it may be applied in order to filter compounds from a large chemical database to be used for docking, as docking is a relatively time consuming process which cannot be easily used for huge number of molecules, clearly not as easy as filtering molecules based on a set of descriptors represented by numbers.

The value of the mean cLogP in our learning set is higher than usual. Values of cLogP thus indicate the preference of being in oil rather than in water. Agonists of this target prefer the hydrophobic regions more than binders at most other targets. We assume this is the case because some of the natural substrates are fatty acids, and the activators tend to be similar to these fatty acids. Fatty acids have long hydrocarbon chains with a polar head group, so the protein binding “pocket” is adjusted to prefer more lipophilic compounds (only three residues can form hydrogen bonds, being deep into the pocket).

A few decisions had to be made before the screening of ISE candidates by docking. Structural features of known protein-ligand complexes are helpful for screening libraries of molecules for discovery (see for example^47^). Apo structures are less relevant due to the conformational changes that frequently accompany ligand binding.

Our approach to docking, which prioritizes on the basis of geometric criteria and not only the energy ones, proved to be successful. This approach refers to the “binding mode” of ligands rather than energies in minimizations or in combinations of Molecular Dynamics and minimizations. Screening by docking traditionally considers the energy criteria to prioritize candidates. Those energies are based on molecular mechanics calculations or empirical evaluations, and do not represent “real” energies due to missing factors, such as lack of information about local dielectric, which determines the strength of electrostatic interactions. Also, Van der Waals atomic parameters are not precise enough to represent the variations in values of single atoms in different local environments.

Therefore, in docking calculations, after collecting the 30 best conformations according to energy scores, we used geometry criteria, based on the known ligand-protein residues interactions in solved crystal complexes. The protein residues that are constantly close to ligands in the crystal complexes were used as the basis for subsequent automatic examination of ligand poses in virtual screening. That is similar to the concept of spatial pharmacophore constraints, but in the “reverse” direction – from protein to ligand rather than the other way. We assign relevant residues from preferentially a few crystal complexes of the same protein with different ligands.

Table S9 presents rankings of selected poses by the energy score of Open Eye’s docking program FRED. Chemgauss3 scoring function^35^ has been employed to measure complementarity of ligand poses with the active site by recognizing shape, H-bonding between ligand and protein and with implicit solvent. Given that each novel agonist has 3 selected poses, one docked in each of the three PDB complexes, there are 42 selected poses for the 14 highest affinity inhibitors. Only 8 out of the 42 (~20%) would be picked at the top if we relied on energy scores alone. Therefore, most of the agonists with EC_50_ < 1 µM could not be predicted as hits without applying our distance criteria.

Our method in this project was to identify residues that interact with at least 70% of the crystallographic ligands. However, as it is customary to use complexes of ligands with proteins for constructing a ligand “pharmacophore”, the reverse may be produced from the same crystal complexes.

We suggest adding consensus geometric criteria for examining the docking of large sets of molecular candidates. The binding of ligands in several crystal complexes of a specific target may be “transformed” into a set of geometry criteria for subsequent docking of unknowns. Applying highly accurate energy calculations is extremely time-consuming in comparison to using geometry criteria. While a single geometrical criterion is not accurate, the use of several such criteria increases accuracy in the same sense as 3D NOESY results of multiple atomic interactions are used to assign molecular conformations. At worst, only a single complex could be used for that purpose, or otherwise, if only a crystal complex of the native protein is known, then docking binders that are known from *in vitro* studies compared to decoys could be used for making distance decisions.

In comparison with studies that applied geometry criteria^48,49^, our geometry criteria are based on consensus decisions that differ from those previous papers by: 1) Protein interacting residues are those that appear in several of the five different complexes (Fig. S8, page S13). The other papers used a single ligand reference or “Tanimoto metric interaction fingerprints” in a single complex. 2) We docked molecules to three different crystal structures in order to compensate for some conformational variations. The other papers do not accommodate any protein flexibility. 3) Screened molecules were picked for experimental validation only if they docked well to all three structures. Thus, we “voted” on the choice of residues to be used for examining binding modes during the docking, “voted” on the crystal complexes to be used for docking and “voted” on the molecules to be sent for *in vitro* testing. For docking, we picked those crystal structures that distinguished well between known *in vitro* agonists and random molecules, as indicated by MCC values. Our approach suggests to deal with the fact that data from crystallographic complexes is “frozen”, while we sample some of the “frozen” states^50^ by using different crystal structures. Our predictions were validated by testing activity of predicted molecules in corresponding cellular assays.

In conclusion, we discovered novel molecular hits and leads for PPAR-δ by applying our combinatorial optimizing algorithm, ISE, followed by docking (by OpenEye’s FRED) and our consensus geometry filters of docking. ISE iteratively picks the best combinations of filters to construct a model for virtual screening libraries consisting of millions of molecules. The combination of ISE with geometry criteria for docking proved to be essential for reducing the number of candidates for experimental testing. ISE in combination with geometric criteria of docking is able to separate the “wheat” from the “chaff” and proves an ability to discover diverse and novel molecules. In combination with docking, we achieved successful ranking and prioritization for molecular bioactivity predictions.

## Methods

Iterative Stochastic Elimination (ISE)^4^: Molecular descriptors (e.g. physico-chemical properties and molecular connectivity) have been used to distinguish between active molecules and inactive (or less active) ones. Filters of 4–5 descriptor ranges are scored by their abilities to identify true positives (the actives, TP) and true negatives (the inactives, TN), as well as false negatives (wrongly identified actives, FN) and false positives (wrongly identified inactives, FP) and the efficiency of each filter is scored by the Matthews Correlation Coefficient (MCC)^34^. Random picking of 4–5 descriptors to construct a filter is the basis for a large combinatorial sampling of filters from which it is possible to assess which descriptors are consistently leading to worst results. Such descriptors are then eliminated and a new iteration proceeds with random construction of filters, while further iterations stop once the total number of potential combinations of descriptors is below a certain threshold which allows all remaining descriptors to construct filters exhaustively, with filters being sorted by their MCC scores and the top most effective filters (up to a value about 20% less than that of the top MCC) constitute our final model.

### Calculating values of physico-chemical properties (descriptors)

Values of physico-chemical properties were calculated by MOE2010^51^ for each molecule in each set (actives, inactives and ENAMINE database). These values were used to validate if the molecules obey the Rule of 5 and the Oprea rule, as well as to calculate the ranges of the applicability domain, and are the basis for producing the ISE model. In the case of the Wu *et al*.^43^ agonists the calculations of properties were performed by MOE2011.

### Calculating ranges of applicability domains

Applicability Domain^33^ is defined by the “chemical space” in which the training set should be developed for model construction. As the training set includes known actives and requires to be “diluted” with many inactives, those should be picked from the same “chemical space”. Some main properties should not bias the classification and therefore should not differ much from main properties of the “actives”.

We apply the requirement that randoms should bear some main similarities to the active molecules: we use applicability domain according to Lipinski’s “rule of 5” properties (values depend on the set of actives): Numbers of H-bond acceptors and H-bond donors, calculated logP and Molecular Weight. Properties were calculated by MOE software (by the descriptors: lip_acc (all N + O), lip_don (all OH + NH), logP(o/w) and molecular weight) for the known agonists and for the random molecules. Mean values and standard deviations of the inhibitors were calculated for each of the properties, and a range of −2σ to 2σ was applied in order to include randomly picked molecules.

### Collecting information from the PDB complexes

The PPAR-δ complexes were collected from the PDB according to the conditions: 1. solved by X-ray crystallography; 2. in complex with a ligand, with published EC_50_; and 3. the resolution is <2.50 Å. Table S4 presents data about the complexes. We summarized these interactions of each complex by using the LigPlot program (data was collected from the complex entries of PDBsum^52^, on the “Ligand” tab). The maximum distance between Donor-and acceptor in Hydrogen bonds (D-A) is 3.3 Å. The maximum distance for VdW interactions is 3.9 Å. We used default distances, as we mentioned above. Ramachandran Plots were produced by PROCHECK^53^.

### Preparation of the PDB complexes and the small molecules, rigid docking and geometrical analysis

For each selected complex of PPAR-δ, some additional preparations in Sybyl-X 2.0^54^ were required. First, we removed all the water molecules. Second, hydrogens were added to the whole protein. Third, we minimized the added hydrogens only. The Force Field was Tripos, the Charge was Gasteiger-Hückel, and the Dielectric Constant used was 4. The minimization was performed in two steps: first, Steepest descent followed by conjugate gradient with 10,000 steps to termination when the difference between successive minimization steps was <0.001 kcal/(mol*Å). A grid for docking was constructed by the MAKE RECEPTOR 3.0.0 (OpenEye) using default parameters^35^.

In preparation for docking, 200 conformations were created for each molecule by the Omega program (OpenEye)^55^. Rigid docking of each was performed by Fred (OpenEye)^35^ using default parameters. For each molecule, 30 ligand poses with best energy scores were picked for analysis. For each pose, distances to the chosen residues were measured, as we mentioned above. A molecule was considered “successfully docked” if at least one of its poses fulfilled the geometrical criteria.

### The criteria for success in docking are

at least one pose out of 30 with distance <3.5 Å to at least 2 of the “crucial to H-bonds” residues, and distance <5 Å to at least 7 of the “important” residues. If a ligand has few successful poses (out of 30) – the pose with the lowest energy score was selected. If none of the poses of a candidate ligand fulfill the criteria – the ligand is rejected.

### Measuring molecular agonist activities

A GAL4-DNA Binding Domain (GAL4-DBD) fusion protein was constructed for each PPAR family member^39^. To assess compound activity, each construct was co-transfected into HEK293T cells (American Type Culture Collection; Manassass, VA) along with the reporter construct, pGL5 (Promega) using Fugene 6 (Promega) as a transfection reagent and the manufacturer’s protocol. An eleven point dilution series of each compound was added to the cells and incubated overnight. Luciferase levels were then determined following the addition of Bright-Glo (Promega) using the manufacturer’s recommendations. EC_50_ values were fitted to sigmoidal curves using four parameter logistic regression (GraphPad). hPPARα/LBD encoding amino acids 175–468 (Genbank accession #NM_001001928), hPPARδ/LBD encoding amino acids 147–441 (Genbank accession #NM_006238) and hPPARγ/LBD encoding amino acids 184–477 (Genbank accession #NM_138712). Each of the 306 candidates that were selected was tested as an agonist of PPAR-α, PPAR-γ and PPAR-δ.

### Diversity of the novel inhibitors

Tanimoto comparisons^32^ between the various sets and among molecules from the same set were made by OpenBabel (FP2 fingerprint)^56^.

## Supplementary information

Discovering highly selective and diverse PPAR-delta agonists by ligand based machine learning and structural modeling

## Data Availability

CSV file with EC_50_ values and computational data will be made available and deposited upon request.
