# Supplementary material for: Discovering highly selective and diverse PPAR-delta agonists by ligand based machine learning and structural modeling
Source: Sci Rep. 2019 Jan 31;9:1106. doi: 10.1038/s41598-019-38508-8 (PMC6355875; doi:10.1038/s41598-019-38508-8)
Supplement: Supplementary file 1 — Discovering highly selective and diverse PPAR-delta agonists by ligand based machine learning and structural modeling [file 41598_2019_38508_MOESM1_ESM.docx]

Supporting Information

**Discovering highly selective and diverse PPAR-delta agonists by ligand based machine learning and structural modeling**

Beny Da’adoosh^1^, David Marcus^1^, Anwar Rayan^1,2,3^, Fred King^4^, Jianwei Che^4,5^, and Amiram Goldblum^1^

*^a^ Molecular Modeling Laboratory, Institute for Drug Research, The Hebrew University of Jerusalem, Israel; ^b^ Institute of Applied Research, Galilee Society, Shefa-Amr, Israel 20200; ^c^ Drug Discovery Informatics Lab, Qasemi-Research Center, Al-Qasemi Academic College, Baka El-Garbiah, Israel 30100; ^d^ Genomics Institute of the Novartis Research Foundation, 10675 John Jay Hopkins Dr., San Diego, CA 92121; ^e^ Dept. of Chem. & Biochem., University of California at San Diego, La Jolla, CA 92037*

*To whom correspondence should be addressed:

[amiramg@ekmd.huji.ac.il](mailto:amiramg@ekmd.huji.ac.il)

jianwei.che@gmail.com

**Supplementary Table S1. Details about the novel agonists, including the names, the ZINC ids, the ENAMINE ids and SMI codes.** The first three molecules are known agonists that were used for the measurements of the novel agonists.

|  | ZINC ID | ENAMINE ID | SMI CODE |
| --- | --- | --- | --- |
| GW501516 | ZINC01549989 | - | CC1=CC(SCC2=C(C)N=C(S2)C3=CC=C(C=C3)C(F)(F)F)=CC=C1OCC(O)=O |
| GW7647 | ZINC03995991 | - | CC(C)(SC1=CC=C(CCN(CCCCC2CCCCC2)C(=O)NC3CCCCC3)C=C1)C(O)=O |
| GW1929 | ZINC03832278 | - | CN(CCOC1=CC=C(C[C@H](NC2=CC=CC=C2C(=O)C3=CC=CC=C3)C(O)=O)C=C1)C4=CC=CC=N4 |
| GNF-0242 | ZINC13015184 | T6811704 / Z107275398 | CC1=CC=CC(OCCNC(=O)COC2=CC=C(CC(O)=O)C=C2)=C1 |
| GNF-8065 | ZINC58163616 | T6757285 / Z2241117324 | COC1=CC=C(CNCC2=CC=C(OC3=CC=CN=C3)C(F)=C2)C=C1O |
| GNF-8501 | ZINC23642332 | T5630172 / Z104661894 | OC1=CC=C(C=C1)N1CCN(CC(=O)NCC2(CCCCC2)N2CCOCC2)CC1 |
| GNF-3632 | ZINC11484008 | T5999328 / Z220348160 | FC(F)(F)COCC1=CC=C(CNC(=O)C2=NNC(=C2)C2=CC=CC=C2)C=C1 |
| GNF-6878 | ZINC03242660 | T0509-5156 / Z56843494 | CCOC1=CC=CC(C=NC2=NC(=CS2)C2=CC=CC(=C2)N=CC2=CC=CC(OCC)=C2O)=C1O |
| GNF-8560 | ZINC09587215 | T5435815 / Z15674334 | CCOC(=O)NC(=O)COC(=O)C1=C2C=CC=CC2=NC2=C1CC(C)CC2=CC1=CC=C(OC)C=C1 |
| GNF-0341 | ZINC12975568 | T6163027 / Z218469708 | CC1=CC=C(C=C1)C(=O)NC1=CC=CC(CNCC2=CC=C(OC(F)(F)F)C=C2)=C1 |
| GNF-6029 | ZINC23257925 | T5894943 / Z2239074203 | ClC1=CC=C(C=C1)C(=O)NC1=CC=CC(CNCC(N2CCOCC2)C2=CC=CS2)=C1 |
| GNF-9820 | Not found | T5307117 / Z15749601 | CC1=CC=C(C=C1)C1=C(N=C(N1)SCCCC(=O)N1CCOCC1)C1=CC=C(C)C=C1 |
| GNF-5295 | ZINC20218763 | T6068856 / Z57728254 | CCC1=CC(C(N2CCN(CCO)CC2)C3=CC=CC(OC)=C3)=C(NC(=O)C4=CC=CC=C4)S1 |
| GNF-5891 | ZINC10292903 | T5466364 / Z146503620 | OC1=CC=CC=C1NC(=O)C1CCN(CC1)C(=O)COC1=CC=C(F)C=C1 |
| GNF-7486 | ZINC09635461 | T5483814 / Z2239061775 | COC1=CC=C(C=C1)N1CCN(CC1)C(=O)CSC1=NN=C(COC2=CC=C(Cl)C=C2C)N1 |
| GNF-6952 | ZINC48278659 | T6631408 / Z827019134 | COC1=CC(CNCC2=NOC(C)=C2)=CC=C1OCC(=O)NC1=CC=C(Br)C=C1 |
| GNF-9448 | ZINC12987589 | T5934377 / Z146502162 | OC1=CC=CC=C1NC(=O)C1CCN(CC1)C(=O)C1=CC=C(OCC2CCCO2)C=C1 |
| GNF-6928 | ZINC28073764 | T6284002 / Z167763814 | OC1=CC=C(C(=O)COC(=O)C2=CC=C(COC3=CC=C4C=CC=CC4=C3)O2)C(O)=C1 |
| GNF-5758 | ZINC13546695 | T0514-3164 / Z2239048625 | COC1=CC(C=C2SC(NC2=O)=NC2=CC=CC=C2)=CC=C1OCC1=CC=CC(=C1)C(O)=O |
| GNF-9594 | ZINC09950634 | T5581560 / Z31332688 | OC(CNCC1=CC=C(F)C=C1)COC1=CC=C(C=C1[N+]([O-])=O)S(=O)(=O)N1CCCC1 |
| GNF-4516 | ZINC03249585 | T0516-5986 / Z2587724546 | CCOC1=CC(C=CC(O)=O)=CC=C1OCC1=CC=C(C=C1)C(C)(C)C |
| GNF-5154 | ZINC13062039 | T6034605 / Z51260512 | COCCCN1C(=O)C2=C(SC3=C2CCCS3)N=C1SCC1=CC=CC(=C1)C(O)=O |
| GNF-7176 | ZINC35336364 | T5700057 / Z191686322 | OC1=CC(=CC=C1[N+]([O-])=O)C(=O)NCCCCOC1=CC=C(F)C=C1 |
| GNF-9057 | ZINC12852236 | T5944667 / Z227718664 | CC1=CC(C(=O)COC2=CC=C(C=C2)C(O)=O)=C(C)N1CCC1=CCCCC1 |
| GNF-0248 | ZINC12916927 | T5958543 / Z227719686 | OC(=O)C1=CC=C(OCC(=O)NC(C2=CC=CC=C2)C2=CC=C(Cl)C=C2)C=C1 |
| GNF-1051 | Not found | T5808401 / Z237518078 | OC(=O)CC1=CSC(SCC2=CC(F)=CC3=C2OC(OC3)C2=CC=CC=C2)=N1 |
| GNF-8208 | ZINC12810508 | T6028636 / Z195978852 | CC(N1CCC(=CC1)C1=CC=C(O)C=C1)C(=O)NC1=CC=C(SC(F)F)C=C1 |
| GNF-4909 | ZINC13097290 | T5501087 / Z95224260 | COC1=CC=C(C=C1CSCC(O)=O)C(=O)C=CC1=C(Cl)N(N=C1C)C1=CC=CC=C1 |
| GNF-1676 | ZINC58161786 | T6753746 / Z954298790 | CCCOC1=C(OCC)C=CC=C1C=CC(=O)C1=CC=C(SCC(O)=O)C(OC)=C1 |
| GNF-9969 | ZINC06080848 | T6124323 / Z25218103 | CC1=CC(C(=O)COC(=O)C2=CC=C(O)C=C2O)=C(C)N1CC1=CC=C(F)C=C1 |


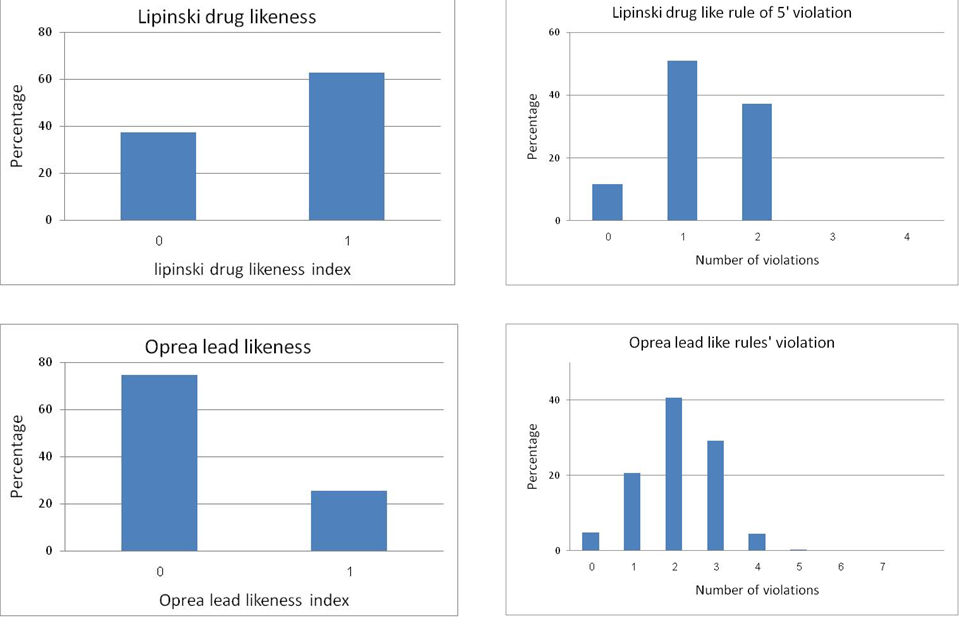


**Supplementary Figure S1. Distribution plots of the Lipinski drug-like rule of 5 (ROF) violations and Oprea lead-like rules' violations for the set of PPAR-δ ligands:** Upper left row, left: Lipinski drug-likeness 0 = agonists that do not obey, 1 = agonists obeying the rule), upper row, right: Distribution of ROF violations – 37% of known agonists violate 2 or more of the 4 rules. Lower row, left: Oprea lead-likeness rules, 0 = agonists that do not obey the rules, 1 = 25% which obey. Lower row, left: Distribution of number of violations of Oprea lead-like rules.


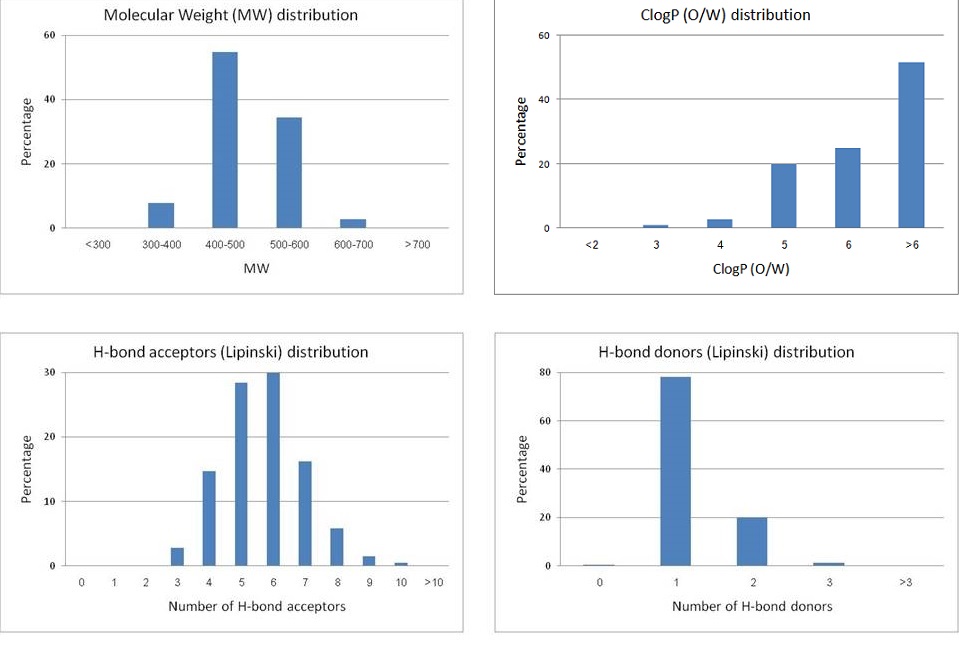


**Supplementary Figure S2. Distribution plots of the four "Rule of 5" descriptors of Lipinski for the set of PPAR-δ agonists.** Upper left and clockwise: percentages of Molecular weights, of CLogP values, of the numbers of H-bond donors, and of the numbers of H-bond acceptors.


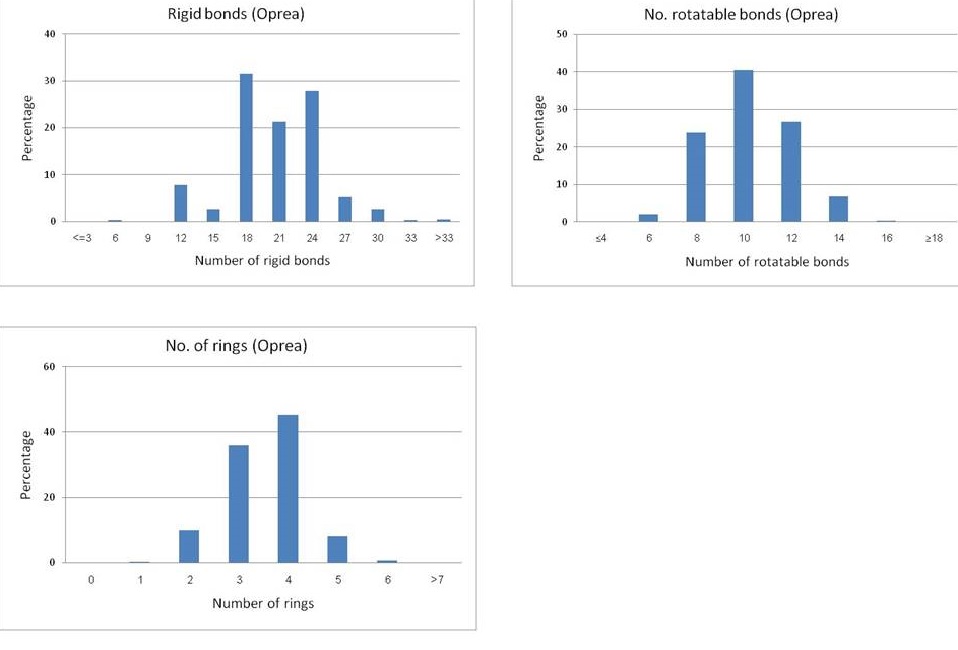


**Supplementary Figure S3. Distribution plots of three additional lead-likeness descriptors of Oprea for the set of PPAR-δ agonists:** Numbers of rigid bonds, Number of rotatable bonds, Numbers of rings.


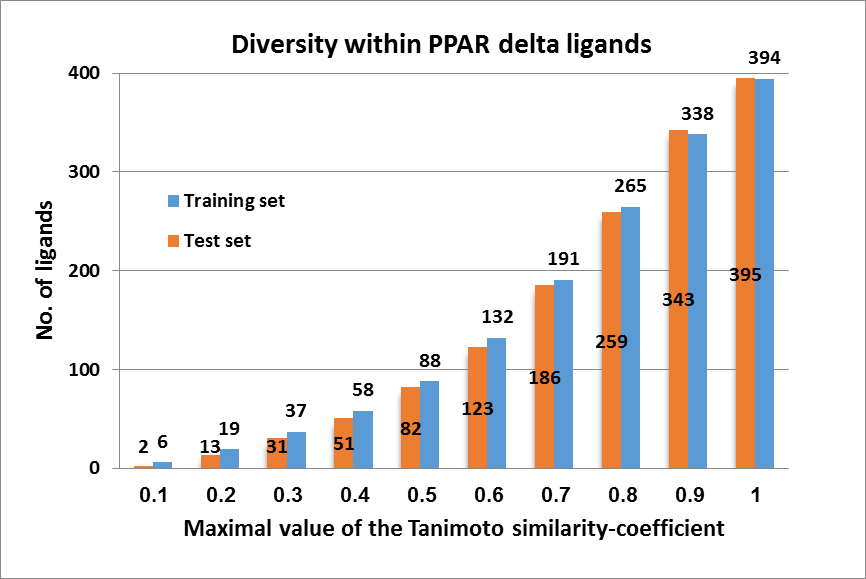


**Supplementary Figure S4. Diversity within the training and test sets of PPAR-δ agonists that were used for modeling.** The number in each bar indicates the active molecules that have remained at each “cutting level” of Tanimoto similarity.


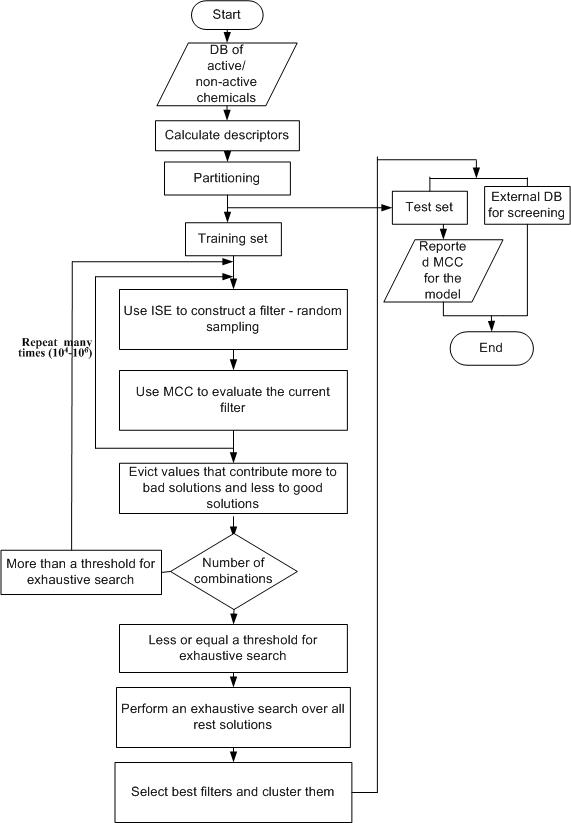


**Supplementary Figure S5. Flowchart of the ISE algorithm.**

**Supplementary Table S2. The most redundant descriptors in the 68 filters, i.e., those that appear more than others among the 272 descriptor ranges (68 filters * 4 descriptor ranges)**. Those filters should also indicate mechanistic aspects of the PPAR-δ agonists. The total number of appearances of descriptors in this table is 205 out of the 272. Considering that 184 descriptors could be randomly distributed among the 68 filters, each descriptor would appear between 1-2 times.

| Descriptor name | % in model (appearances) | Meaning |
| --- | --- | --- |
| a_count | 99 (67) | Number of atoms (including hydrogens) |
| Q_VSA_PPOS | 32 (22) | Total positive polar VDW surface area |
| Q_VSA_PNEG | 31 (21) | Total negative polar VDW surface area |
| Q_VSA_FPOS | 24 (16) | Fractional positive VDW surface area |
| a_Ni | 21 (14) | Number of Iodine atoms |
| vsa_acc | 18 (12) | Sum of VDW surface areas of pure H-bond acceptors * |
| Q_VSA_FHYD | 12 (8) | Fractional hydrophobic VDW surface area |
| BCUT_PEOE_2 | 10 (7) | In adjacency matrix of 1/sqrt(bij), where bij is the formal bond order between atoms i and j, the resulting eigenvalues are sorted and the 2/3-ile is reported ** |
| KierA3 | 10 (7) | Third kappa shape index *** |
| SMR_VSA7 | 10 (7) | Sum of the VDW surface area, but only in atoms with molar refractivity > 0.56 |
| opr_brigid | 9 (6) | The number of the rigid bonds according to Oprea **** |
| PEOE_VSA-2 | 9 (6) | Sum of the VDW surface area, but only in atoms with partial charges of [-0.15,-0.10) |
| PEOE_VSA_POL | 9 (6) | Total polar VDW surface area |
| TPSA | 9 (6) | Topological Polar Surface area |

* This is an approximation. In addition, not counting acidic atoms or atoms that are both donors and acceptors; ** The diagonal values in the matrix are the partial charges according to the partial equalization of orbital electronegativities (PEOE) method, and see reference [^1^](#_ENREF_1) for more details *** For reference see [^2^](#_ENREF_2) **** For reference see [^3^](#_ENREF_3)


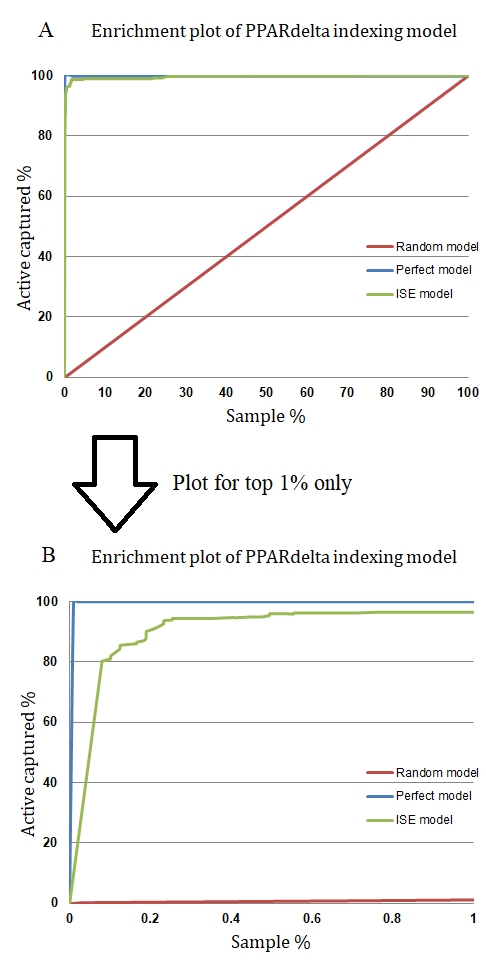


**Supplementary Figure S6.** **Enrichment plot (a) and Enrichment plot of the highest indexed 1% chemicals (b).** For each active molecule in the set, we have 1000 inactive molecules (mix ratio 1:1000). A “Perfect model” is a model that predicts all the active molecules in the 0.01% of the top ranked molecules.


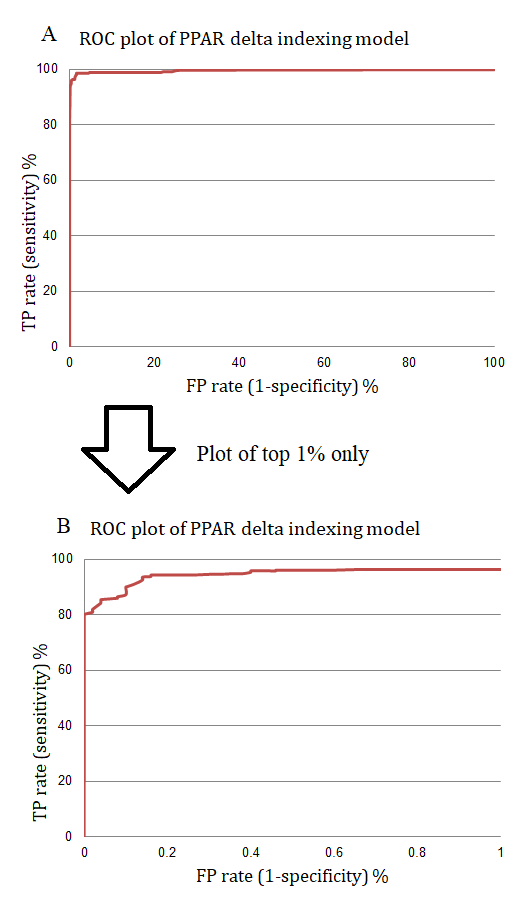


**Supplementary Figure S7. A receiver operating characteristic (ROC) curve (a) and ROC curve of the highest indexed 1% chemicals (b).** The area under the ROC curve is above 0.98, revealing highly accurate and efficient model. The “TP rate” (sensitivity) is the percent of the True Positives among both True Positives and False Positives (i.e, the active agonists that are ranked among all the molecules in the top), while the “FP rate” (specificity) is the percent of the True negatives among both True Negatives and False Negatives (i.e., the inactive molecules that aren’t ranked among all the molecules in the top).

**Supplementary Table S3. Numbers of ENAMINE molecules that achieved different scores by the ISE model.**

| % of commercial molecules | No. of commercial molecules over than | % True Positives | MBI Threshold |
| --- | --- | --- | --- |
| 100 | 1563097 | 100 | -25 |
| 20.47 | 320041 | 99 | -20 |
| 10.91 | 170624 | 99 | -15 |
| 5.91 | 92395 | 99 | -10 |
| 2.71 | 42404 | 99 | -5 |
| 1.19 | 18604 | 99 | 0 |
| 0.51 | 8111 | 96 | 5 |
| 0.16 | 2491 | 95 | 10 |
| 0.04 | 711 | 87 | 15 |
| 0.008 | 129 | 75 | 20 |
| 0 | 0 | 36 | 25 |


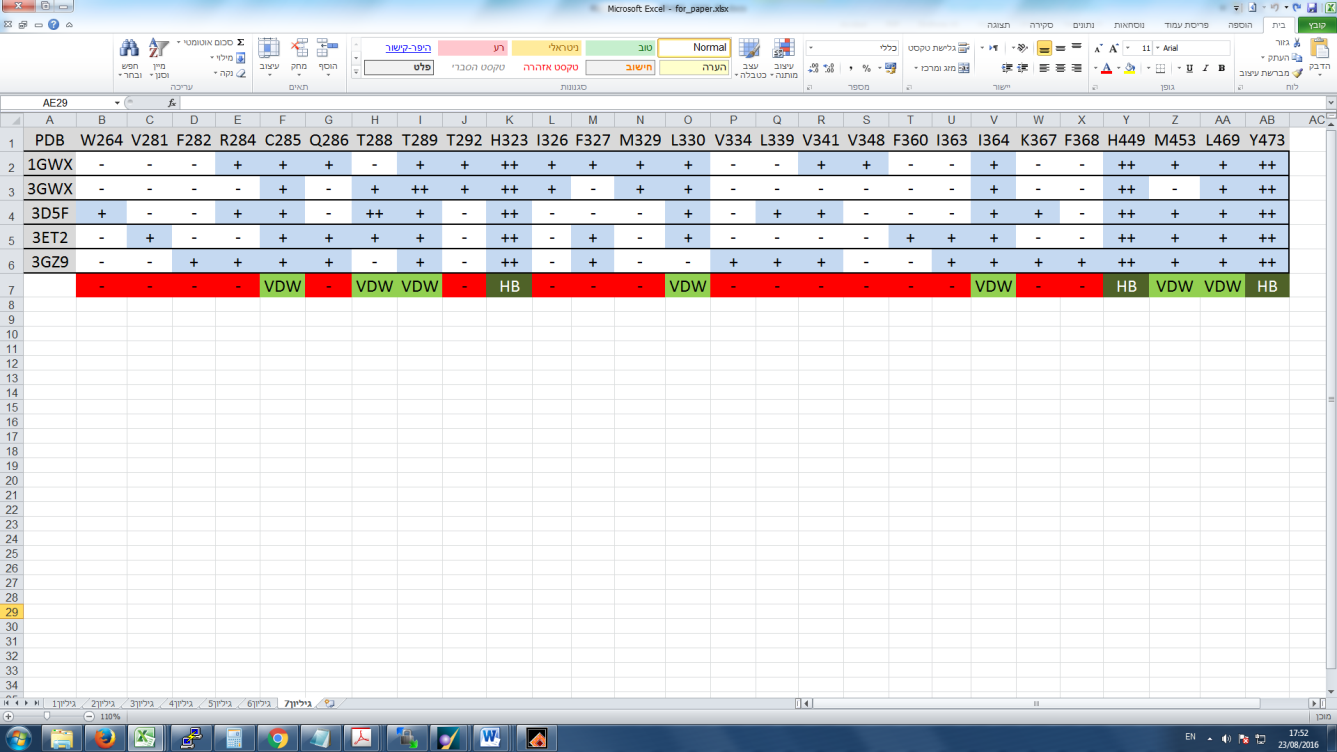


**Supplementary Figure S8. Analysis of the PPAR-δ residues interacting with ligands in crystal complexes.** For each residue, the bottom line indicates its use in subsequent docking experiments - either HB (indicating that this residue is important for Hydrogen bonding with the ligand), or VdW – residue is important for VdW interactions. Others have not been considered in the analysis of docking results.


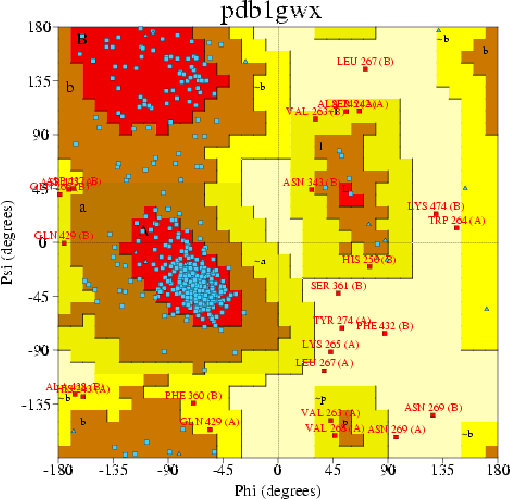

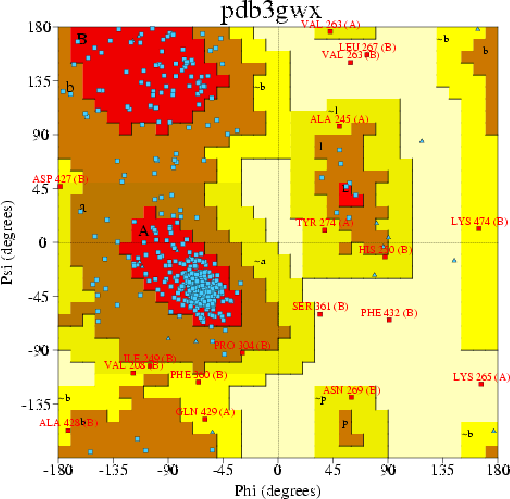

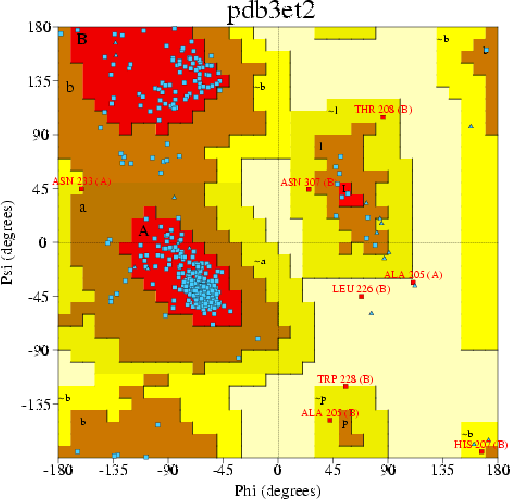

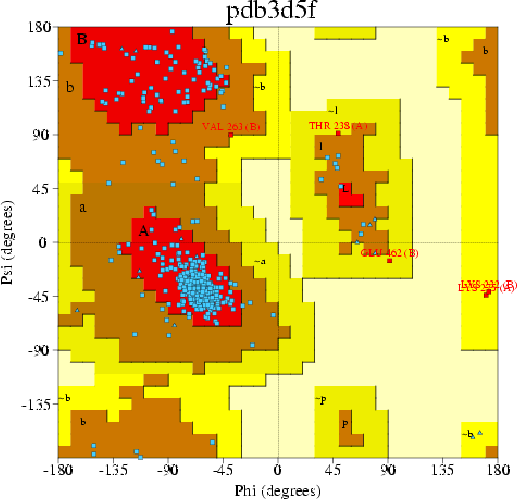


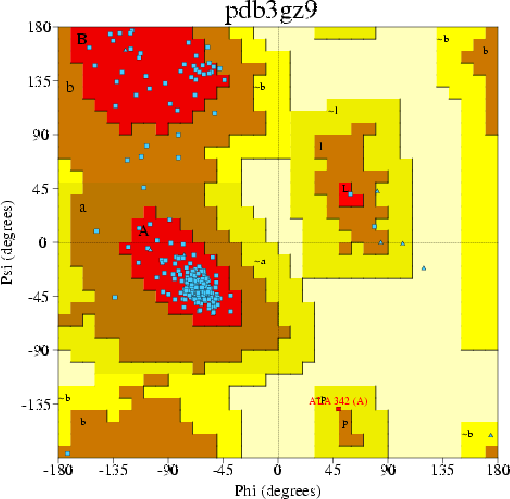


**Supplementary Figure S9. Ramachandran Plots of the PDB complexes 1GWX; 3GWX; 3ET2; 3D5F; 3GZ9.** In contrast to 1GWX, 3GWX and 3ET2, there are not residues on the outliers in the complexes 3D5F and 3GZ9

**Supplementary Table S4. Geometrical data as well as energy score of the selected poses in the re-docking**. None of the poses in the case of 3ET2's re-dockings fulfilled the criteria.

| The complex | Number of "crucial to H-bonds" residues with distance < 3.5Å to the selected pose | Number of "important" residues with distance < 5Å to the selected pose | Energy score (Fred) |
| --- | --- | --- | --- |
| 1GWX a | 10 | 3 | -123.12 |
| 1GWX b | 10 | 2 | -115.35 |
| 3GWX a | 10 | 3 | -77.00 |
| 3GWX b | 10 | 3 | -87.16 |
| 3D5F a | 10 | 3 | -100.93 |
| 3D5F b | 10 | 3 | -103.60 |
| 3GZ9 | 9 | 2 | -69.19 |

**Supplementary Table S5. Details about the complexes from the PDB.**

| PDB | 1GWX | 3GXW | 3D5F | 3ET2 | 3GZ9 |
| --- | --- | --- | --- | --- | --- |
| Num. of chains | Homodimer | Homodimer | Homodimer | Homodimer | Monomer |
| Resolution (Å) | 2.5 | 2.4 | 2.2 | 2.24 | 2.0 |
| Ramachandran plot | 10 Outliers | 5 Outliers | No Outliers | 2 Outliers | No Outliers |
| Re-docking | Successful | Successful | Successful | Failure | Successful |
| Docked Agonists* (out of 135) | 84/97 | 83/54 | 116/109 | 94/84 | 112 |

* The first number is the number of the docked agonists for chain A, and the second for chain B.

**Supplementary Table S6. Ranges of Lipinski properties based on ~1200 known agonists of PPAR-δ*.** The above ranges are "applicability domain": it is expected that in a "learning set" in which we mix known actives with "assumed inactives" we should pick the "decoys" (inactives) to be in the same "properties domain" as the actives. Thus, for this docking, 1000 molecules, which are within the applicability domain of the actives, were picked randomly from Enamine commercially available database.

| Property | Average | Standard Deviation | Range |
| --- | --- | --- | --- |
| Hydrogen bond acceptors (lip_acc) | 5.71 | 1.31 | 3.08-8.33 |
| Hydrogen bond donors (lip_don) | 0.26 | 0.53 | 0-1.33 |
| LogP (o/w) | 6.13 | 1.33 | 3.47-8.80 |
| Molecular Weight | 477.24 | 59.32 | 359-596 |

* Values are averages +/- 2 Standard deviations.

**Supplementary Table S7. EC50 values of 64 molecules, which are not agonists for PPAR-δ, but the other PPARs.** For molecule structures – look at Fig S10.

| Name | EC_50_ PPAR-δ (µM) | EC_50_ PPAR-α (µM) | EC_50_ PPAR-γ (µM) |
| --- | --- | --- | --- |
| T0519-9550 | 10.000 | >10 | 0.277 |
| T6970553 | 10.000 | 7.638 | 0.348 |
| T5981093 | 10.000 | 4.811 | 0.949 |
| T6009822 | 10.000 | >10 | 1.191 |
| T6752465 | 10.000 | 4.555 | 1.205 |
| T6121200 | 10.000 | 7.404 | 1.287 |
| T5367928 | 10.000 | 2.201 | 1.610 |
| T5486524 | 10.000 | 1.379 | 1.701 |
| T6004945 | 10.000 | 8.832 | 2.048 |
| T5592463 | 10.000 | >10 | 2.060 |
| T5628375 | 10.000 | >10 | 2.283 |
| T5501687 | 10.000 | 1.599 | 2.784 |
| T5284148 | 10.000 | >10 | 4.145 |
| T6986392 | 10.000 | 3.132 | 4.718 |
| T5483748 | 10.000 | 3.332 | 5.369 |
| T5492492 | 10.000 | 4.446 | 5.839 |
| T5807049 | 10.000 | >10 | 6.004 |
| T6291645 | 10.000 | >10 | 6.022 |
| T5335018 | 10.000 | >10 | 6.265 |
| T5330889 | 10.000 | >10 | 6.309 |
| T0519-8799 | 10.000 | >10 | 6.442 |
| T6000213 | 10.000 | >10 | 6.870 |
| T6754162 | 10.000 | >10 | 6.916 |
| T0511-1281 | 10.000 | >10 | 7.008 |
| T6773671 | 10.000 | 8.761 | 7.013 |
| T0519-8792 | 10.000 | >10 | 7.055 |
| T6610369 | 10.000 | >10 | 7.183 |
| T5569503 | 10.000 | >10 | 7.203 |
| T5349357 | 10.000 | >10 | 7.214 |
| T5389454 | 10.000 | >10 | 7.290 |
| T5470599 | 10.000 | 6.772 | 7.471 |
| T5373680 | 10.000 | 2.356 | 7.774 |
| T5662965 | 10.000 | >10 | 7.842 |
| T5375021 | 10.000 | >10 | 8.056 |
| T6272279 | 10.000 | >10 | 8.112 |
| T6711938 | 10.000 | >10 | 8.356 |
| T5287095 | 10.000 | >10 | 8.617 |
| T5234137 | 10.000 | >10 | 8.749 |
| T0518-4840 | 10.000 | 2.477 | 8.754 |
| T5932895 | 10.000 | >10 | 8.759 |
| T6623510 | 10.000 | >10 | 9.050 |
| T0516-9261 | 10.000 | 7.466 | 9.254 |
| T5292851 | 10.000 | >10 | 9.416 |
| T6875127 | 10.000 | 4.698 | 9.904 |
| T6025157 | 10.000 | 1.198 | >10 |
| T6720039 | 10.000 | 1.645 | >10 |
| T5424541 | 10.000 | 1.857 | >10 |
| T6018263 | 10.000 | 4.643 | >10 |
| T6192882 | 10.000 | 4.784 | >10 |
| T6046611 | 10.000 | 5.979 | >10 |
| T6785533 | 10.000 | 6.823 | >10 |
| T5936449 | 10.000 | 7.699 | >10 |
| T5901519 | 10.000 | 7.754 | >10 |
| T5872814 | 10.000 | 7.852 | >10 |
| T5224095 | 10.000 | 8.204 | >10 |
| T6129595 | 10.000 | 8.311 | >10 |
| T5465128 | 10.000 | 8.320 | >10 |
| T6288701 | 10.000 | 8.374 | >10 |
| T6632619 | 10.000 | 8.829 | >10 |
| T5380005 | 10.000 | 8.881 | >10 |
| T0515-1394 | 10.000 | 8.911 | >10 |
| T6016677 | 10.000 | 9.017 | >10 |
| T5936399 | 10.000 | 9.551 | >10 |
| T5941878 | 10.000 | 9.939 | >10 |

**Supplementary Figure S10. Novel agonists of PPARs, but not of PPAR-δ.**

**Supplementary Table S8. MBI for each of the molecules that were discovered by Wu at el.**

| Agonist | EC_50_ |
| --- | --- |
| Compound1 | 13.80 |
| Compound2 | 18.30 |
| Compound3 | 17.58 |
| Compound4 | 18.67 |
| Compound5 | 17.62 |
| Compound6 | 16.91 |
| Compound7 | 13.10 |
| Compound8 | 17.28 |
| Compound9 | 17.62 |
| Compound10 | 16.91 |
| Compound11 | 17.95 |
| Compound12 | 17.95 |
| Compound13 | 18.67 |
| Compound14 | 18.67 |
| Compound15 | 17.58 |
| Compound16 | 18.67 |


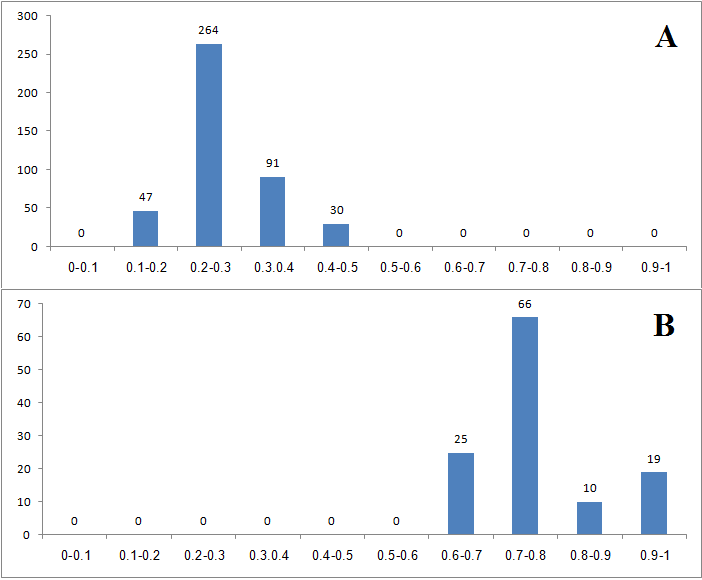


**Supplementary Figure S11. Distribution of Tanimoto values for the novel agonists of Wu et al:** A. Comparison vs. the novel agonists in this paper. B. Comparison of these novel agonists among themselves.

**Supplementary Table S9. The energy ranking of the selected poses agonists with highest affinities is shown, for each of the three PDB complexes used for docking.** Only 8 of the 42 poses were ranked best by energy.

| Name | 3D5F A | 3D5F B | 3GZ9 |
| --- | --- | --- | --- |
| GNF-0242 | 9 | 7 | 1 |
| GNF-8065 | 1 | 1 | 7 |
| GNF-8501 | 16 | 12 | 17 |
| GNF-3632 | 14 | 1 | 10 |
| GNF-6878 | 5 | 2 | 1 |
| GNF-8560 | 11 | 17 | 5 |
| GNF-0341 | 6 | 16 | 3 |
| GNF-6029 | 6 | 9 | 8 |
| GNF-9820 | 24 | 25 | 8 |
| GNF-5295 | 1 | 6 | 7 |
| GNF-5891 | 8 | 3 | 2 |
| GNF-7486 | 4 | 4 | 11 |
| GNF-6952 | 3 | 7 | 2 |
| GNF-9448 | 1 | 3 | 1 |

**Results and discussion of docking the top 13 candidate activators**

Docking of the 13 molecules that displayed low nanomolar activation (4-19 nM) was performed as mentioned in the main text. OpenEye's FRED was employed as described and each of these molecules was docked, as all others, to three different crystal structures of PPAR-delta.

As required for any of the ~2500 molecules (the top ISE candidates out of 1.56 millions) to be selected by the docking results, a pose must have at least 7 Van der Waals bonds (out of 10 in our analysis of the crystals) and 2 hydrogen bonds (out of 3). We present the selected poses for those 13 molecules in each of the 3 crystals in table S10 and in figure S12. Except for two cases (GNF-6878 and GNF-5891), the poses are very similar for all others.

There is a major difference between our results and those of the crystal structures with respect to the groups that display major interactions with PPAR-delta. In the crystal structures, the chemical group that interacts with the amino acid triad (H323, H449, Y473) is carboxylic acid (present most probably as carboxylate). That is the result in all PPAR-δ X-ray complexes that served us for learning the essential interactions (1GWX, 3GWX, 3D5F, 3ET2 and 3GZ9) as well as in those that were published later (18 PDB IDs in the paper of Wu et al. – ref. 43 in the main text, or 3TKM complexed to GW0742). Out of the 13 top novel lead activators that were discovered computationally, only the most potent one GNF-0242 (EC50 = 4 nM) interacts with the triad bya carboxylic moiety. None of the other leads have a carboxylic acid as part of their scaffolds, so the leads carry different chemical groups that fulfill the demands for interactions (Fig. S13).

Furthermore, it should be noted that among the novel leads there is substantial diversity: the chemical groups that interact with the triad are different among those 13 leads. We attribute that diversity to the main character of the ISE discovery process, as it uses physic-chemical properties and no chemical groups. This issue was discussed in the main text.

Finally, we present two of our novel leads, GNF-8065 and GNF-8501, together with 7 crystal structures of PPAR-delta – ligand complexes, in figure S14. These leads display greater potency than one of the activators that is currently in pharmaceutical developments, GW0742 (EC_50_ = 30 nM) as well as better potency than most of the agonists in the paper of Wu et al. (three are activators with EC_50_ values of 9, 17 and 19nM, three others with 42, 54, and 61nM, while the other 10 have larger EC_50_ values over 100 nM up to 1.96 micromolar. See ref. 43 in the main text).

Thus, the discovery of the novel chemical groups that can interact with the triad, might be a new milestone in our efforts to achieve a therapeutic agent for PPAR-δ as a medicinal target.

**Table S10. The number of the Van der Waals interactions and the number of H-bonds for each of the novel leads according to docking results into three PPAR-δ x-ray structures.** The interactions were determined by the distances from the ten selected residues (see the main text)

|  | 3D5F A | | 3D5F B | | 3GZ9 A | |
| --- | --- | --- | --- | --- | --- | --- |
| Lead | VdW interactions | H bonds | VdW interactions | H bonds | VdW interactions | H bonds |
| GNF-0242 | 9 | 3 | 10 | 3 | 9 | 3 |
| GNF-8065 | 10 | 2 | 10 | 2 | 10 | 2 |
| GNF-8501 | 9 | 3 | 9 | 2 | 10 | 2 |
| GNF-3632 | 9 | 2 | 10 | 3 | 10 | 2 |
| GNF-6878 | 9 | 2 | 9 | 3 | 10 | 3 |
| GNF-8560 | 10 | 2 | 9 | 3 | 10 | 2 |
| GNF-0341 | 9 | 2 | 9 | 2 | 9 | 2 |
| GNF-6029 | 9 | 2 | 10 | 2 | 10 | 2 |
| GNF-9820 | 10 | 2 | 10 | 2 | 10 | 2 |
| GNF-5891 | 10 | 3 | 10 | 2 | 10 | 2 |
| GNF-5295 | 10 | 2 | 9 | 3 | 8 | 2 |
| GNF-7486 | 10 | 2 | 10 | 2 | 10 | 2 |
| GNF-6952 | 10 | 2 | 10 | 2 | 10 | 2 |


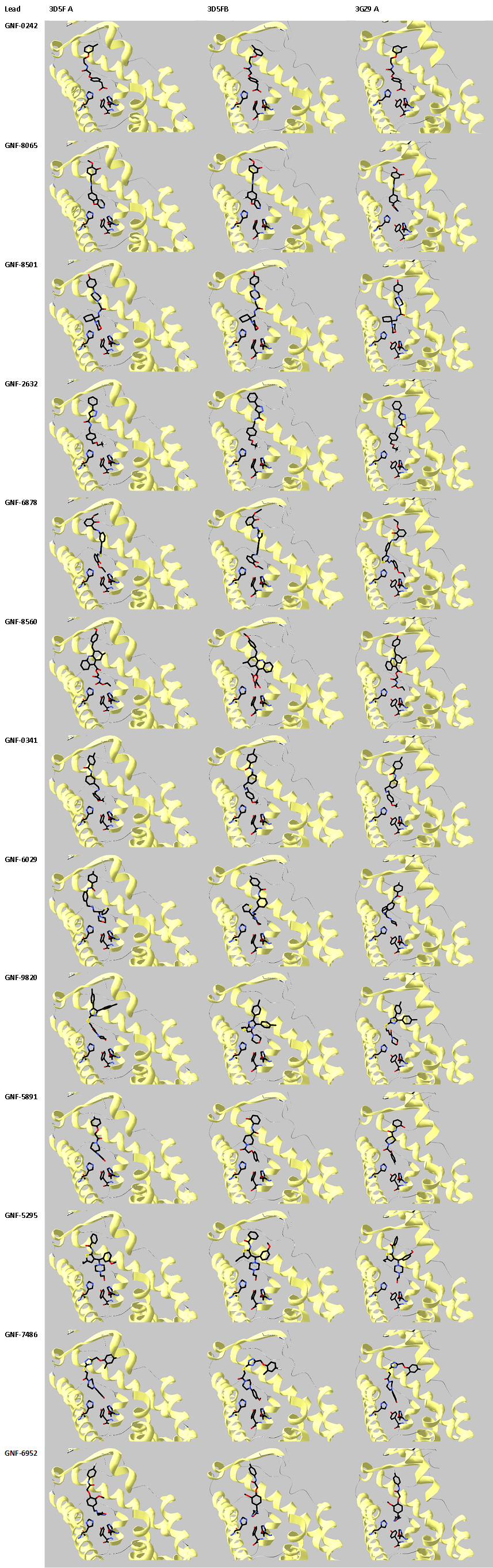


**
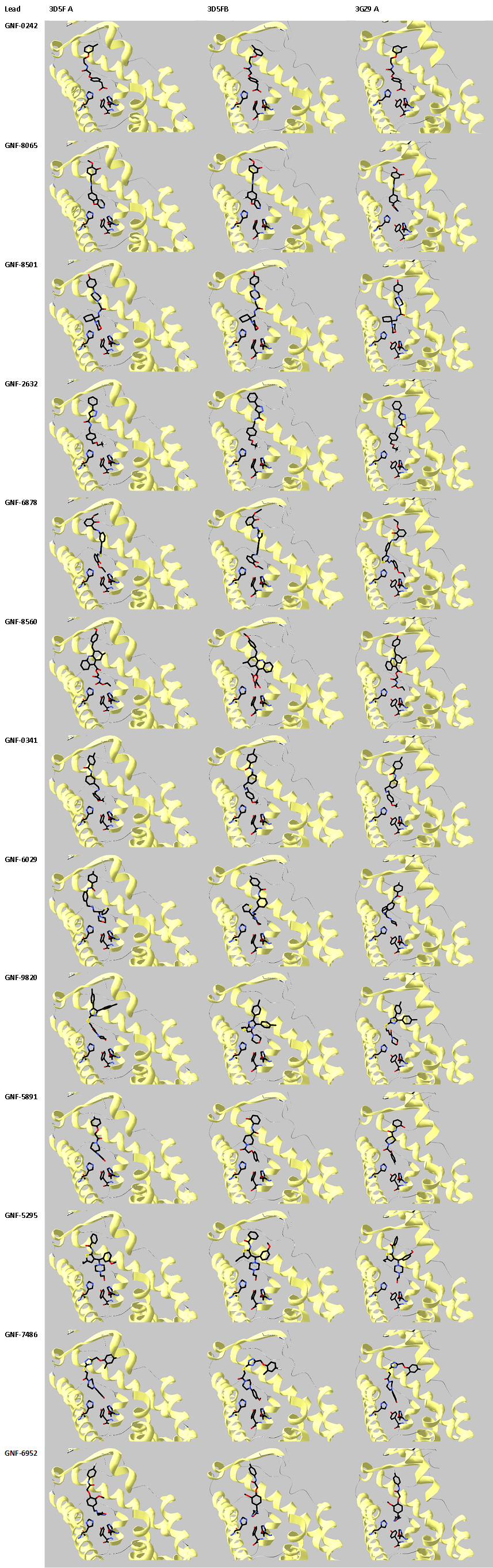
**

**
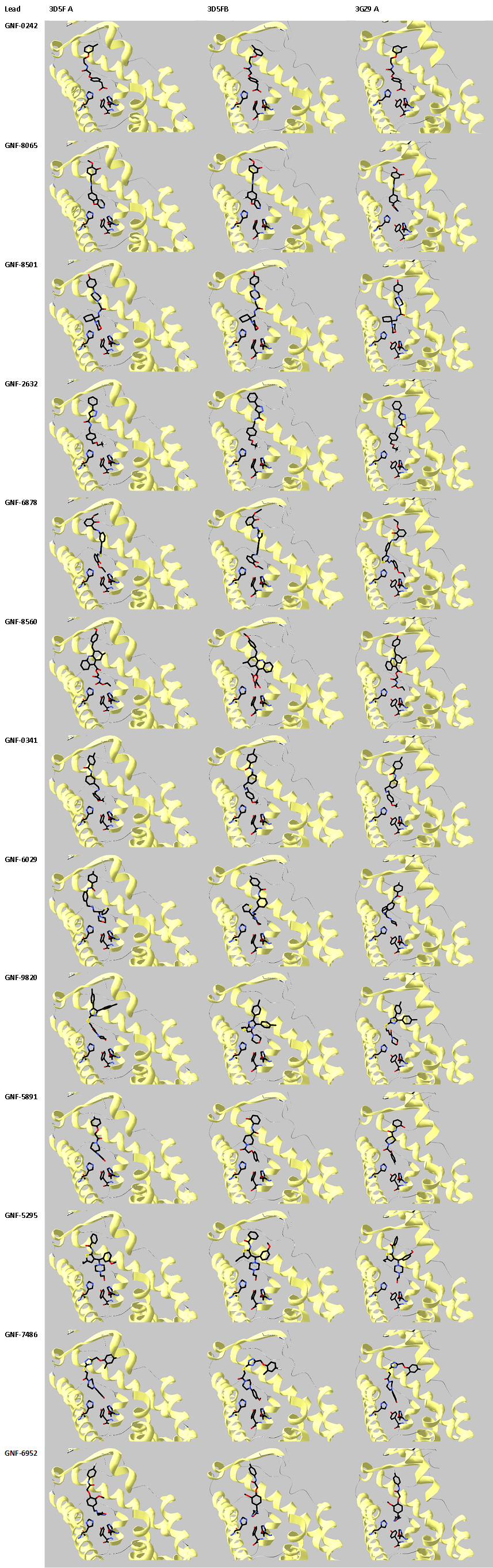
 Fig. S12. The poses of the 13 top agonists (see figure 1 in the main text) in docking to PPAR-δ x-ray structures.** The triad (H323, H449, Y473) is presented at the bottom of the binding site. Figures were prepared by Swiss PDB Viewer.

**Fig. S13. Fragments that interact with the triad in our top agonists.** GNF-6878, includes the same benzendiol derivative at both ends of the molecule and one of these is found twice near the triad, the other one appears once. In the case of GNF-5891 the fragment that contacts with 3D5F (chain B) and 3GZ9 is presented. The labels are the names of the original molecules (including ENAMINE codes, and EC_50_ values).


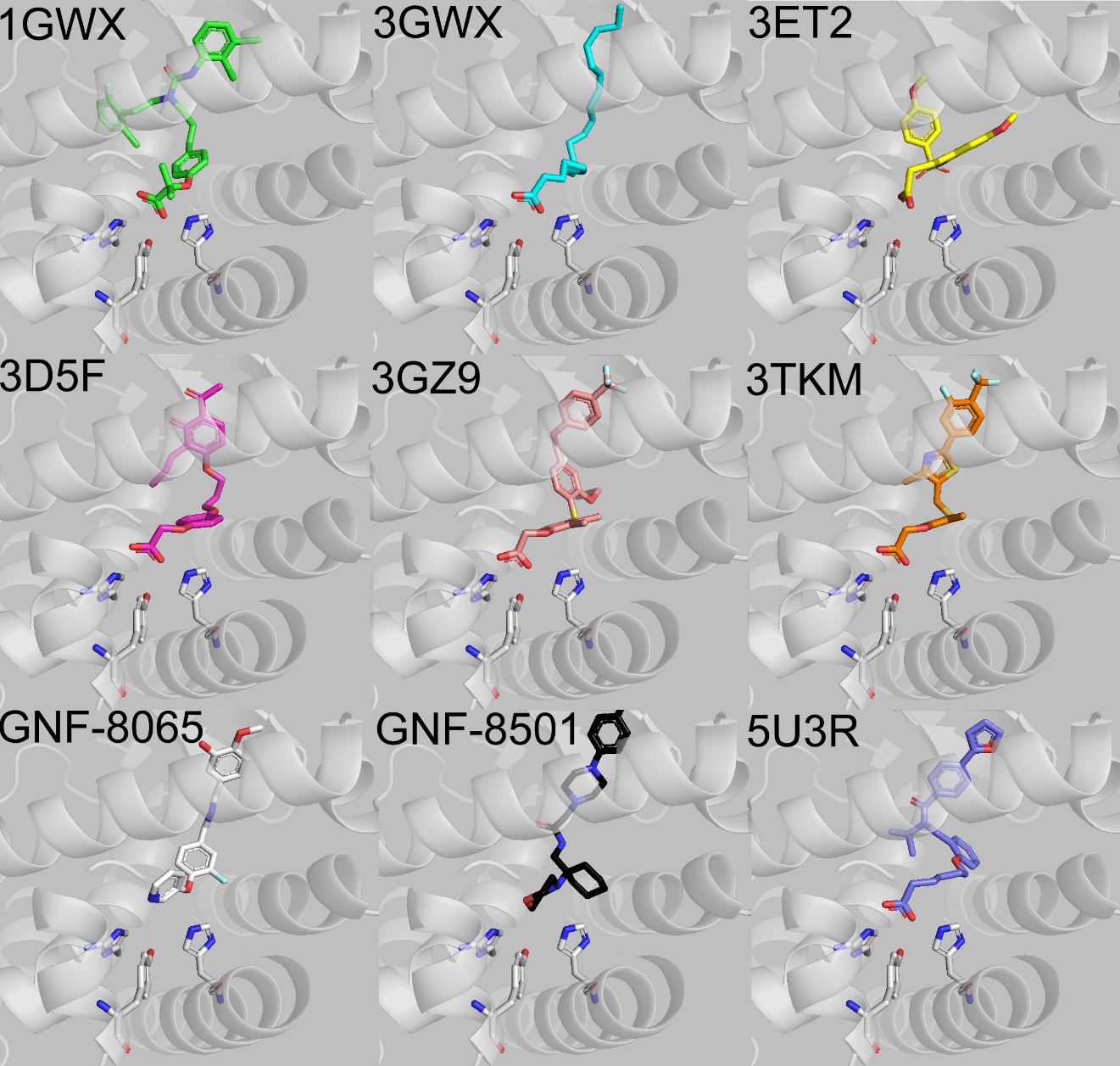


**Fig. S 14. Presenting poses of agonists of PPAR-δ in published crystal structures, together with two predicted poses by computational docking (to 3D5F chain B) of our novel agonists GNF-8065 and GNF-8501, showing all results near the triad (H323, H449, Y473).** Only in the case of our novel leads , new chemical groups are found to interact with the triad. Individual structures of ligand positions were prepared by Pymol and assembled for this figure.

**References**

(1) Oprea, T. I. *J Comput Aid Mol Des* **2000**, *14*, 251.

(2) Hall, L. H.; Kier, L. B. In *Reviews of Computational Chemistry 2*; Lipkowits, K. B., Boyd, D. B., Eds.; VCH: New York, 1991, p 362.

(3) Pearlman, R. S.; Smith, K. M. *Perspect Drug Discov* **1998**, *9-11*, 339.
